# The initiation knot is a signaling center required for molar tooth development

**DOI:** 10.1242/dev.194597

**Published:** 2021-04-29

**Authors:** Isabel Mogollón, Jacqueline E. Moustakas-Verho, Minna Niittykoski, Laura Ahtiainen

**Affiliations:** 1 Cell and Tissue Dynamics Research Program, Institute of Biotechnology, University of Helsinki, 00014, Finland; 2 Organismal & Evolutionary Biology Research Program, University of Helsinki, 00014, Finland

**Keywords:** Cell division, Migration, Embryonic development, Tooth, Signaling center, Wnt, Shh

## Abstract

Signaling centers, or organizers, regulate many aspects of embryonic morphogenesis. In the mammalian molar tooth, reiterative signaling in specialized centers called enamel knots (EKs) determines tooth patterning. Preceding the primary EK, transient epithelial thickening appears, the significance of which remains debated. Using tissue confocal fluorescence imaging with laser ablation experiments, we show that this transient thickening is an earlier signaling center, the molar initiation knot (IK), that is required for the progression of tooth development. IK cell dynamics demonstrate the hallmarks of a signaling center: cell cycle exit, condensation and eventual silencing through apoptosis. IK initiation and maturation are defined by the juxtaposition of cells with high Wnt activity to *Shh*-expressing non-proliferating cells, the combination of which drives the growth of the tooth bud, leading to the formation of the primary EK as an independent cell cluster. Overall, the whole development of the tooth, from initiation to patterning, is driven by the iterative use of signaling centers.

## Abstract (precis)

**Summary:** During tooth morphogenesis, transient thickening of the epithelium in the diastema anterior to the first developing molar is an early signaling center, the molar initiation knot (IK), which is required for the progression of mammalian molar tooth development.

## INTRODUCTION

In recent years, advances in 3D and live tissue imaging have brought new understanding of the cell-level behaviors that contribute to the highly dynamic stages of morphogenesis in ectodermal organs, such as hair and teeth (Ahtiainen et al., 2014, 2016; Devenport and Fuchs, 2008; Kim et al., 2017). Despite shared morphological characteristics and conserved signaling (Biggs and Mikkola, 2014; Jernvall and Thesleff, 2000), it is becoming evident that signaling cues are interpreted into diverse cellular behaviors depending on the context, thereby defining different organ shapes and sizes already at early stages of organogenesis. Morphogenesis in ectodermal organs is regulated by epithelial signaling centers, which form sequentially in specific spatiotemporal patterns and govern cell behaviors via secreted factors, including hedgehog (Hh), Wnt, fibroblast growth factor (Fgf) and bone morphogenetic protein (Bmp) family members (Dassule and McMahon, 1998; Jernvall and Thesleff, 2000; Yu and Klein, 2020).

Teeth have long served as a model organ for studying mechanisms of embryonic development in tissue interactions and genetic regulation (Jernvall and Thesleff, 2000). Mice have two tooth types: large, ever-growing incisors and multicuspid molars. Organogenesis in teeth is initiated at embryonic day (E)11 with an epithelial thickening called the dental lamina. This resolves into separate domains for incisor and first molar primordia, with a toothless diastema in between. The instructive potential resides initially in the epithelium and shifts to the mesenchyme at the bud stage. Epithelial budding starts at E12.5, followed by mesenchymal condensation leading to a mature bud at E13.5 (Jernvall and Thesleff, 2000). The molar primary enamel knot (pEK) signaling center appears at E13.5 in the late bud-stage epithelium and matures into the enamel organ in the cap stage at E14.5 (Munne et al., 2009). The pEK is silenced by apoptosis and sequentially followed by pairs of secondary EK (sEK), which regulate cusp patterning (Jernvall et al., 1994). Fate-mapping studies have shown that the pEK clonally contributes to the buccal sEK, but may not contribute to the lingual counterpart (Du et al., 2017).

The cell-level events in tooth development are now beginning to be understood, assisted by emerging live tissue microscopy techniques (Mogollon and Ahtiainen, 2020). The early developmental stages of tooth development in particular have been challenging to investigate with conventional developmental biology approaches because of the shortage of reporters to follow the dynamic cellular events in intact tissue. Recently, we identified a novel epithelial signaling center in the early developing incisor, called the initiation knot (IK), that drives local cell proliferation for epithelial budding (Ahtiainen et al., 2016). The incisor IK shares transcriptional signatures with the incisor EK, which forms without clonal contribution from the IK (Ahtiainen et al., 2016; Du et al., 2017; Li et al., 2016a). Although the molar placode and EKs are known to share molecular markers, a signaling center in the molar similar to that in the incisor has not been reported. However, previous studies using expression and histological analyses of molar morphogenesis prior to budding have interpreted a transient epithelial thickening in the diastema anterior to the first developing molar as evidence for the presence of vestigial premolar teeth lost during murine evolution (Prochazka et al., 2010).

To resolve the early events of molar morphogenesis, we used confocal fluorescence whole-mount live tissue imaging to elucidate the cellular and molecular dynamics of signaling centers and how they shape the tooth bud. We show that an IK signaling center is established in the molar placode and remains an integral functional part of the developing bud. The molar IK arises by the juxtaposition of cells with high canonical Wnt activity to *Shh*-expressing G_1_/G_0_-phase cells. Early molar growth is dependent on the IK signaling center and interference in the function of this signaling center, either mechanically, by laser ablation, or with specific modulators of relevant signaling pathways, abrogates bud proliferative growth and progression of tooth development. The IK positions the tooth in the growing mandible and is silenced by apoptosis as the pEK arises independently to drive further growth of the bud. Thus, the cellular and molecular dynamics of the IK signaling center control tooth development earlier than was previously thought.

## RESULTS

### A molar initiation knot is established in the placode and early bud in G_1_/G_0_ cells expressing signaling center markers

Cell cycle exit is an early hallmark of ectodermal placodes (Ahtiainen et al., 2014, 2016). The Fucci fluorescent cell cycle reporter system allows direct real-time follow-up of the progress of the cell cycle in individual cells in the developing tissue: when the cell is in G_1_/G_0_ phase, the nucleus emits red fluorescence; upon transition to S/G_2_/M proliferative phase, the cell nucleus emits green fluorescence. We used confocal fluorescence microscopy of whole-mount mandibles of the Fucci cell cycle indicator transgenic mouse to characterize G_1_/G_0_ cell distribution in the developing molar. Transgenic Shh^GFP/+^ (Harfe et al., 2004) expression was used to identify signaling centers from E11.5 to E13.5, and EpCam immunofluorescence staining was used to visualize the epithelium.

**Fig. 1.**
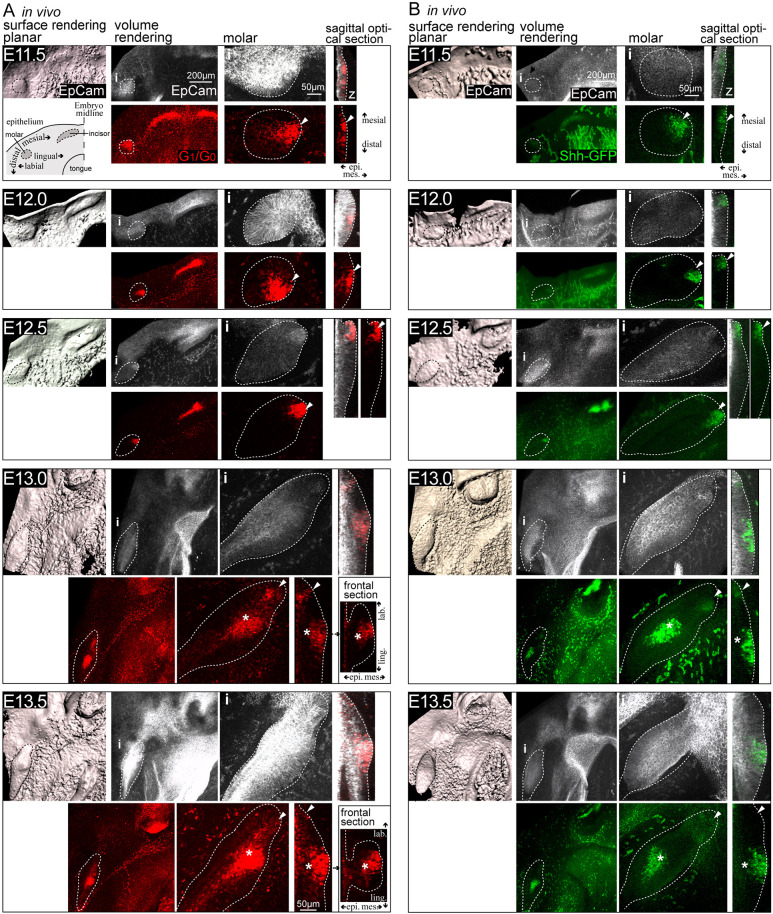
**A molar initiation knot is established in the molar placode and early bud in G_1_/G_0_ cells positive for signaling center markers.** (A,B) Confocal fluorescence images of mouse embryonic mandibles of the cell cycle indicator Fucci for G_1_/G_0_ phase nuclei (red) and the signaling center marker Shh^GFP^ (green). Images show immunofluorescence staining of the epithelium (EpCam, gray; the tooth epithelium perimeter is indicated by dotted lines), early G_1_/G_0_ focus (arrowheads) and presumptive pEK (asterisks). Images show a planar view from the mesenchyme toward the epithelium; i indicates the location of the areas shown at higher magnification on the right; z indicates sagittal optical sections. (A) G_1_/G_0_ cells were present throughout the dental lamina and the molar placode as the incisor and molar resolved into separate domains at E11.5. At E12.5, G_1_/G_0_ cells formed a focus mesially in the molar early bud. This focus remained close to epithelial surface mesiolingually. At E13.0, G_1_/G_0_ cells corresponding to the presumptive pEK emerged in the tip of the bud and condensed by E13.5. (B) Shh^GFP^ signaling center marker showing expression corresponding to G_1_/G_0_ foci throughout placode and bud morphogenesis, and in the emerging pEK.

At E11.5, G_1_/G_0_ phase cells were distributed throughout the dental lamina (Fig. 1A). By E12.5, the G_1_/G_0_ cells were located mesially in the mature placode/early bud. At E13.0, the G_1_/G_0_ focus remained in the mesiolingual part of the bud, close to epithelial surface, and a new focus of G_1_/G_0_ cells appeared distally deep in the invaginating bud, in the presumptive pEK area. By E13.5, the early G_1_/G_0_ focus was lost, with only a few cells remaining (Fig. 1A). In parallel, G_1_/G_0_ cells corresponding to the pEK area emerged.

The Shh^GFP^ and Fucci G_1_/G_0_ reporters could not be combined because this often resulted in abnormal development of the craniofacial structures. However, the Shh^GFP^ reporter showed expression in the same areas as the G_1_/G_0_ foci throughout morphogenesis (Fig. 1B): GFP^+^ cells were present throughout the placode at E11.5. At E12.5, they were located at the mesiolingual side of the early bud, close to the epithelial surface. By E13.5, the GFP signal had disappeared almost completely from the early G_1_/G_0_ focus and appeared in the presumptive pEK area. Digoxigenin (DIG) *in situ* hybridization with a probe specific for *Shh* in Fucci G_1_/G_0_ reporter mandibles showed exact colocalization of *Shh* with the Fucci reporter (Fig. S1A). Quantification of G_1_/G_0_ cells in different developmental stages showed a similar decrease in the G_1_/G_0_ and corresponding *Shh*-expressing cell number in the early focus from E12.5 to E13.5 (Fig. S1B). For further functional analyses, we verified that the bud growth and G_1_/G_0_-*Shh* cell distribution in *ex vivo*-cultured whole-mount explants were similar to those seen *in vivo* (Fig. S1C,D).

To further verify the signaling center identity of the Fucci G_1_/G_0_ *Shh*-expressing cells, we used two known reporters for signaling centers: the Fgf20^βGal/+^ and the fluorescent canonical Wnt signaling reporter TCF/Lef:H2B-GFP mouse models. Immunofluorescence staining for β-galactosidase (βGal) in Fgf20^βGal/+^;Fucci G_1_/G_0_ embryos showed marker colocalization in the E11.5 early G_1_/G_0_ focus, through early bud stage (E12.5-E13.0) and in the pEK at E13.5 (Fig. S1E). The TCF/Lef:H2B-GFP reporter was detected in partially overlapping areas; however, a proportion of the G_1_/G_0_ cells remained distinct and did not show TCF/Lef:H2B-GFP reporter activity (Fig. S1F).

Together, these data confirmed the identity of the initial molar placode G_1_/G_0_ focus and corresponding focus in the mesiolingual part of the developing molar bud epithelium as a signaling center. This early signaling center appeared prior to the pEK and, thus, we called this signaling center a molar IK.

### The molar IK is a functional signaling center driving molar bud proliferative growth

Next, we studied cell proliferation in the developing molar. In the incisor, budding occurs via cell proliferation regulated by non-proliferative signaling centers (Ahtiainen et al., 2016), whereas cell rearrangements and migration together with Shh-driven proliferation have been proposed as mechanisms for molar bud invagination (Dassule and McMahon, 1998; Prochazka et al., 2015; Li et al., 2016b). To dissect the IK contribution to the molar bud, we first studied cell proliferation with Fucci S/G_2_/M and G_1_/G_0_ cell cycle indicators in fixed whole-mount mandibles. The Fucci S/G_2_/M reporter was previously shown to label the proliferating cell population in tooth specifically, consistent with 5-ethynyl-2′-deoxyuridine (EdU) labeling (Ahtiainen et al., 2016). We then imaged whole-mount mandible explant cultures using live tissue confocal microscopy, which allowed us to follow the developing bud at single-cell resolution.

**Fig. 2.**
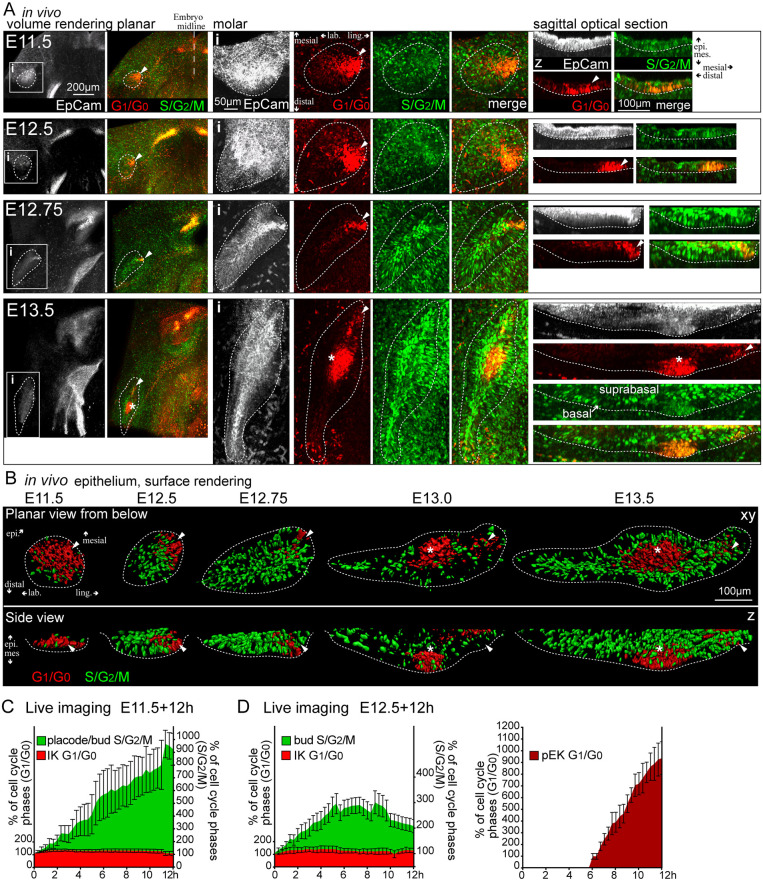
**The molar IK is a functional signaling center driving budding via proliferation.** (A) Confocal fluorescence images of whole-mount explants showing Fucci G_1_/G_0_ nuclei (red), S/G_2_/M nuclei (green) and epithelium/tooth perimeter (EpCam, white, dotted line). i indicates the areas shown at higher magnification on the right and z indicates sagittal optical sections. IK are indicated by arrowheads and pEK by asterisks. Initially, S/G_2_/M cells were seen throughout the oral epithelium and, by E12.5, in the early bud posterior to the IK in both basal and suprabasal populations. IK and pEK cells remained in G_1_/G_0_. (B) Surface rendering of the nuclei in G_1_/G_0_ and S/G_2_/M cell cycle phases in the developing molar placode/tooth bud epithelium. (C) Quantification of cell cycle phases in the IK and early bud with live imaging of E11.5+12 h molars. Data are mean±s.e.m. (*n*=5). (D) Quantification of cell cycle phases in the IK, emerging pEK and tooth bud with live imaging of E12.5+12 h molars Data are mean±s.e.m. (*n*=3).

Live tissue confocal microscopy of the Fucci G_1_/G_0_ reporter for visualization of the molar IK and pEK cells, and of the K17-GFP reporter to follow the shape of the epithelial bud from E12.5+16 h, confirmed that the IK cells remain an integral part of the developing bud (Fig. S2A). Observing proliferation patterns in high resolution with the Fucci reporters at fixed, carefully staged, time points showed that, during early initiation at E11.5, the placode comprised G_1_/G_0_ cells, and S/G_2_/M cells were evenly distributed throughout the oral epithelium (Fig. 2A,B). By E12.5, a majority of tooth early bud cells were in S/G_2_/M, apart from the IK in the mesial part of the bud. From E12.5 to E12.75, there was a sudden increase in S/G_2_/M cells throughout the bud epithelium in both basal and suprabasal populations (Fig. 2A,B). At E13.5, S/G_2_/M cells were present in the bud and surrounding the pEK area, although a few IK G_1_/G_0_ cells remained. Quantification of cell number showed few proliferative cells in the placode at E11.5 (Fig. S2B). At E12.5, during the initiation of budding, there was a threefold increase in S/G_2_/M cell number and a further twofold increase at E13.0, with similar cell numbers in *ex vivo* cultures (Fig. S2B). Proliferation was concurrent with bud elongation and invagination.

We next analyzed the contribution of individual cells in each cell population to the growing bud. Quantification of cell cycle phases with live imaging from E11.5+12 h molars showed a constant G_1_/G_0_ cell number in the IK (Fig. 2C). A burst of cell proliferation from 4 h onwards was seen in the emerging bud. This specific increase in cell proliferation was confined to the tooth bud, and the contributions of G_1_/G_0_ and S/G_2_/M cells remained constant in the oral epithelium (Fig. 2D). When we followed individual IK cells through the cell cycle from E11.5+12 h, we observed new G_1_/G_0_ cells appearing in the IK, whereas two cells showed nuclear fragmentation (Fig. S2D, Movie 1). None of the followed G_1_/G_0_ cells in the IK re-entered the cell cycle. Live imaging from the early bud stage onwards from E12.0+12 h showed that more bud cells entered S/G_2_/M (Fig. S2E, Movie 2). There was a respective increase in cell divisions throughout the bud as it grew. When we followed individual proliferating cells, of the 126 original S/G_2_/M cells followed, 25% went through cytokinesis and divisions were observed throughout the bud (Fig. S2E, Movie 2). Some IK cells showed nuclear fragmentation and were lost, whereas the remaining IK cells stayed in G_1_/G_0_. Quantification of cell cycle phases from E12.5+12 h showed that the number of molar IK G_1_/G_0_ cells decreased slightly (Fig. 2D). The bud S/G_2_/M population continued to expand, plateaued after 6 h and then declined. This coincided with the appearance of the first G_1_/G_0_ cells contributing to the pEK. In both the E11.5 and E12.5 stages, an increase in proliferation was specific to the tooth bud (Fig. S2C).

Therefore, we conclude that the molar IK is a functional signaling center that regulates proliferation in tooth bud invagination and growth. The molar bud is formed by localized cell proliferation.

### IK ablation arrests progression of tooth development

To confirm that the IK drives molar bud growth and is necessary for the progression of tooth development, we ablated the IK at different developmental stages by microsurgery and laser ablation. When the placode was microsurgically removed at E11.5 and the tissue cultured for 24 h, no G_1_/G_0_ condensate was observed in the diastema and the epithelium remained flat (Fig. S3A,C). Microsurgical removal of the IK at E12.5 similarly arrested tooth growth, whereas development on the untreated side proceeded to the bud stage, with the emerging pEK present (Fig. S3B,C).

**Fig. 3.**
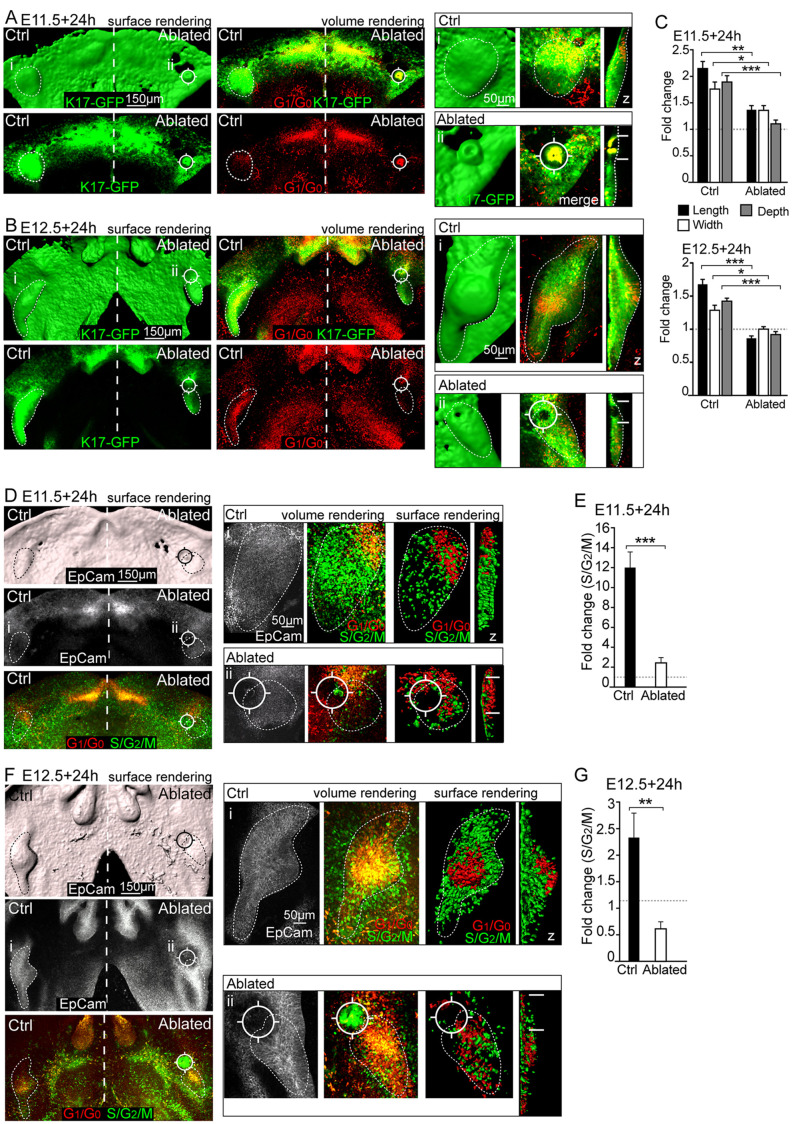
**Laser ablation of the IK arrests molar bud growth.** Confocal fluorescence images of whole-mount explant cultures of K17-GFP epithelium, Fucci S/G_2_/M nuclei (green), Fucci G_1_/G_0_ (red) and epithelium (EpCam, gray). Tooth placode/bud epithelium is indicated by dotted lines, i indicates the areas shown at higher magnification on the right and z indicates sagittal optical sections. The IK was laser ablated (position marked by a viewfinder symbol ¤ and by horizontal lines in *z*-plane views) at E11.5 or E12.5 followed by 24 h culturing. (A) Laser ablation of IK G_1_/G_0_ cells in early placode-stage epithelium (E11.5) abrogated epithelial invagination and growth. On the control side, tooth invagination proceeded normally. (B) Early bud stage (E12.5) ablation completely arrested bud invagination and elongation. (C) Bud dimensions of ablated and control molars at E11.5+24 h and E12.5+24 h. Data show the fold change over E11.5 and are mean±s.e.m.; *n*_E11.5+24h_=9, *n*_E12.5+24h_=8, Mann–Whitney *U*-test; *P≤0.05, **P≤0.01 and ***P≤0.001. (D) Laser ablation of the IK in the Fucci S/G_2_/M model at E11.5 resulted in loss of bud cell proliferation. Physiological pattern of S/G_2_/M cells in the adjacent oral epithelium confirmed good tissue health. (E) Quantification of proliferating cells in E11.5+24 h molars. Data are mean±s.e.m.; *n*=8, Mann–Whitney *U*-test, ***P≤0.001. (F) Bud cell proliferation and consequently bud growth were similarly abrogated in E12.5 ablated molars. (G) Quantification of proliferating cells in E12.5+24 h molars. Data show the fold change over E12.5 and are mean± s.e.m.; *n*=8, Mann–Whitney *U*-test, *P*≤0.01.

For a more-targeted approach, we next removed the IK G_1_/G_0_ cells at E11.5, E12.5 and E12.75 with laser ablation, followed by 24 h culture in K17-GFP and in Fucci whole-mount mandibles. Laser ablation of the IK G_1_/G_0_ cells in the early placode stage (E11.5) epithelium abrogated epithelial invagination and tooth development, whereas development progressed normally in the non-ablated control (Fig. 3A,C) and in the control that was ablated right next to the tooth bud leaving the signaling center intact (Fig. S3D). Ablation at early bud stage (E12.5) similarly completely arrested bud invagination and elongation, and inhibited progression of tooth development (Fig. 3B,C). In addition at this stage, the control side and the control laser-ablated tooth bud developed normally (Fig. S3E). Ablation at E12.75 at a more-developed bud stage also arrested growth; however, a small cluster of G_1_/G_0_ cells was observed in the bottom of the bud facing the mesenchyme in the area where the pEK would emerge (Fig. S3F). To observe whether the arrested growth resulted from abrogated proliferation, we laser ablated the IK in the Fucci model. Correspondingly, ablation at E11.5 arrested invagination (Fig. 3D), accompanied by a loss of cell proliferation in the bud (Fig. 3E). The persistence of S/G_2_/M cells in the immediately adjacent oral epithelium and mesenchyme confirmed good tissue health in non-ablated tissue (Fig. 3SD). Cell proliferation and, consequently, bud growth were similarly abrogated in E12.5 molars where the IK was ablated (Fig. 3F,G).

These experiments demonstrate that the molar IK is a functional signaling center that drives cell proliferation, thereby regulating tooth bud growth. Thus, the IK is necessary for the progression of tooth development.

### The IK remains an integral part of the developing molar and does not contribute cells to the pEK

We next used whole-mount live tissue imaging to track individual cell movement in the different cell populations in the placode and bud stage to dissect whether dynamic cell rearrangements contribute to molar bud formation. Signaling centers show canonical Wnt activity and we used the TCF/Lef:H2B-GFP reporter to visualize Wnt-active cells together with the Fucci G_1_/G_0_ reporter to track signaling center cells. We further imaged the Fucci G_1_/G_0_ reporter with the S/G_2_/M reporter to follow the proliferating bud cell population.

**Fig. 4.**
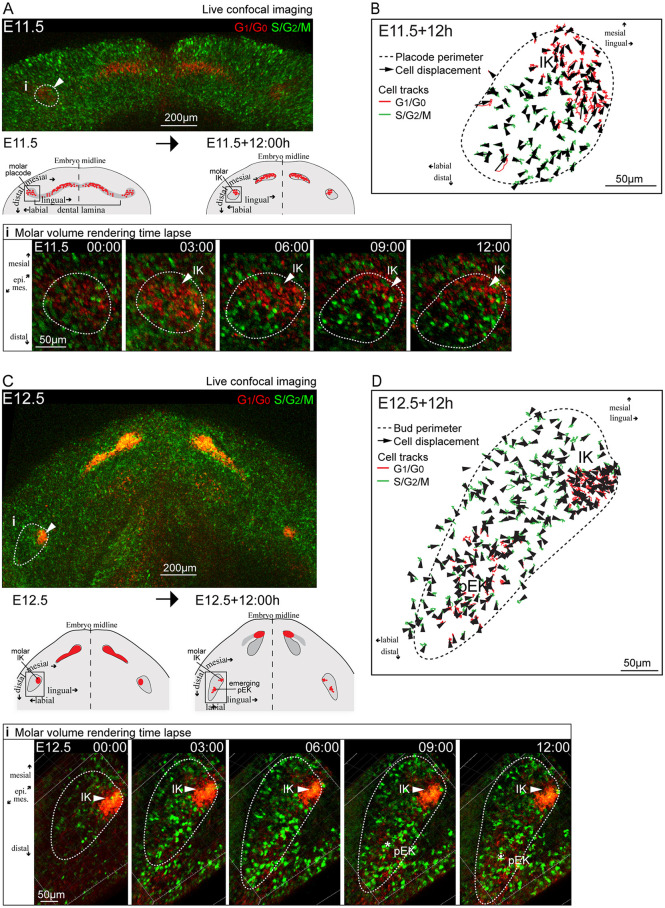
**The IK remains an integral part of the tooth and does not contribute cells to the pEK, which arises independently.** (A) Still images of live tissue time-lapse confocal fluorescence imaging from the placode stage at E11.5 (whole mandible) and molar close-up (i) at E11.5+12 h (E11.5, +3 h, +6 h, +9 h and +12 h) showing Fucci G_1_/G_0_ (red) and S/G_2_/M (green) cells. Arrowheads indicate the IK position. Dotted lines indicate the tooth placode/bud epithelium. (B) Tracks and displacement vectors of individual Fucci G_1_/G_0_ and S/G_2_/M cells in the E11.5+12 h molar culture. (C) Still images of Fucci G_1_/G_0_ (red) and S/G_2_/M (green) reporter live imaging from the early bud stage at E12.5 to +12 h. i indicates the area shown at higher magnification in the molar volume rendering time lapse images. Arrowheads indicate the IK and asterisks indicate the pEK. (D) E12.5+12 h cell tracks and displacement of individual Fucci G_1_/G_0_ (red) and S/G_2_/M (green) cells.

Initially at E11.5, G_1_/G_0_ cells were localized in the molar placode (Fig. 4A, Movie 3), similar to that observed in fixed samples (Fig. 1A). During the next 12 h, more cells differentiated, entered G_1_/G_0_ and were distributed mesiolingually in the maturing placode. Tracking of all Fucci reporter-positive cells showed that IK cells moved toward the mesial front area of the bud and remained an integral part of the bud. In contrast, bud S/G_2_/M cells stayed mostly in place (Fig. 4B). At E11.5, Wnt activity was seen throughout the dental lamina, visualized by high TCF/Lef:H2B-GFP reporter fluorescence intensity (Wnt^Hi^) (Fig. S4A, Movie 4). The molar placode IK G_1_/G_0_ cells specifically localized to the peripheral border formed by dental lamina Wnt^Hi^ cells. There was some overlap of G_1_/G_0_ and Wnt^Hi^ signals, but the G_1_/G_0_ cells mostly remained as a distinct subgroup (Fig. S4A,B, Movie 4). More G1 cells were recruited to the IK during the next 12 h and showed directional movement toward the dental lamina Wnt^Hi^ cells. The dental lamina Wnt^Hi^ cells and bud TCF/Lef:H2B-GFP+ cells remained non-motile (Fig. S4A,B, Movie 4).

Tracing cell movement in the molar IK and the emerging pEK from E12.5+12 h showed that IK cells remained mesiolingually close to the bud surface (Fig. 4C,D, Movies 5, 6). We did not detect contribution from either IK G_1_/G_0_ cells or Wnt^Hi^ to the pEK (Fig. 4C,D, Movie 6, Fig. S4B,C). The pEK arose deep in the bud, without clonal contribution from the IK (Fig. 4C,D, Movie 6, Fig. S4C,D). In addition at this stage, the S/G2/M cells showed little movement with no obvious orientation (Fig. 4C,D, Movie 5), and no contribution of cells from oral epithelium to bud growth was detected.

### Cell condensation and active directional cell migration drive molar IK maturation

Our live imaging experiments showed that IK cells reorganize dynamically during placode/bud maturation. To define the significance of this for IK maturation, we quantified IK cell condensation and analyzed whether the movements involved active cell migration.

**Fig. 5.**
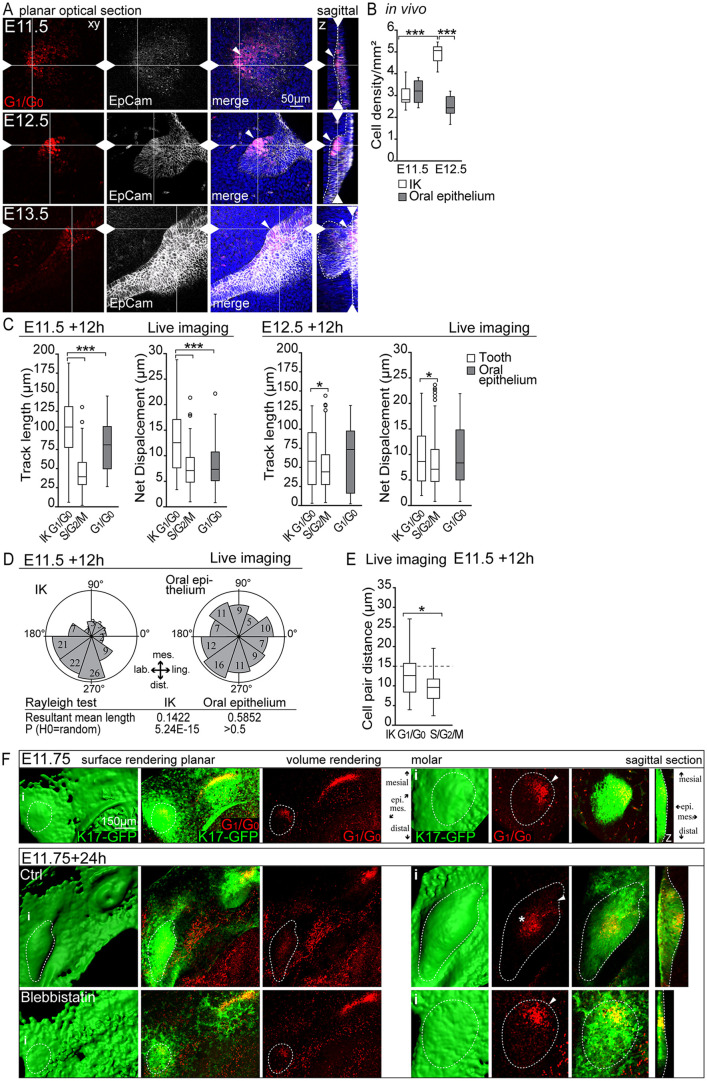
**Cell condensation and active directional cell migration drive molar IK maturation.** (A) Confocal fluorescence images of Fucci G_1_/G_0_ (red) molars, cell borders (EpCam, white, dotted line) and nuclei (Hoechst, blue). (B) Quantification of IK and oral epithelial cell density (plots represent minimum, 25th percentile, median, 75th percentile and maximum values; *n*_placode/bud_=*n*_oral_=10, Mann–Whitney *U*-test, ***P≤0.001). (C) Quantification of cell track length and net displacement in molar placode/bud and oral epithelium at E11.5+12 h (plots represent minimum, 25th percentile, median, 75th percentile and maximum values; *n*_G1/G0 IK_=149, *n*_G1/G0 oral_=45, *n*_S/G2/M_=146, Mann–Whitney *U*-test, ***P≤0.001) and E12.5+12 h (data are mean±s.e.m., *n*_G1/G0 IK_=90, *n*_G1/G0 oral_=51, *n*_S/G2/M_=317, Mann–Whitney *U*-test, *P≤0.05). (D) Quantification of molar IK and oral epithelial cell movement angles E11.5+12 h (data are mean±s.e.m., *n*_IK cells_=*n*_oral epithelial cells_=95, Rayleigh test: H_0_=random, IK *P*≤0.001, oral *P*>0.05). (E) Pairwise comparison of molar IK G_1_/G_0_ and bud S/G_2_M cell positions (plots represent minimum, 25th percentile, median, 75th percentile and maximum values; *n*_pairs_=40, Mann–Whitney *U*-test, *P≤0.05). (F) Confocal fluorescence images of Fucci G_1_/G_0_ nuclei (red) and K17-GFP (epithelium, green) reporter. Tooth placode/bud epithelium is indicated by dotted lines, IK is indicated by arrowheads and pEK by an asterisk. i indicates the area shown at higher magnification on the right and z indicates sagittal optical sections. E11.75 cultures were treated at the time of most active IK G_1_/G_0_ cell movement with blebbistatin for 24 h to inhibit actomyosin-based cell motility. Blebbistatin treatment repressed IK G_1_/G_0_ cell condensation and arrested bud morphogenesis.

We first measured cell density in EpCam-stained fixed whole-mount samples. Initially, at E11.5, G_1_/G_0_ cells were more dispersed; at E12.5, they had condensed and retained this density until E13.5 (Fig. 5A,B). Oral epithelial cells did not show a similar condensation. Quantification of cell density showed that condensation was specific to IK cells, with a significant increase in density from E11.5 to E12.5 compared with the oral epithelium (Fig. 5B).

To study whether IK condensation is achieved through active cell migration, we followed the movement of individual cells by live imaging at E11.5 and E12.5+12 h. Tracking showed active migration of the molar IK G_1_/G_0_ cells at both time points. We quantified the overall track length and net displacement in the different cell populations and, at E11.5+12 h, a significant difference in IK G_1_/G_0_ cells was observed: these migrated more compared with both oral epithelial G_1_/G_0_ and tooth bud S/G_2_/M cells (Fig. 5C). At E12.5+12 h, IK G_1_/G_0_ cells still showed a longer mean track length in the bud compared with S/G2/M cells (Fig. 5C). Quantification of IK G_1_/G_0_ cell displacement angles at E11.5+12 h showed a distinct orientation towards the mesiolingual side of the forming placode/bud, whereas oral epithelial cells showed a random orientation (Fig. 5D). If the IK cells were only pushed by the proliferating cells, they should remain together with their neighbors. Therefore, we confirmed active cell migration by following pairs of IK G_1_/G_0_ and bud S/G_2_/M cells that were initially in close proximity (distance ≤15 µm). The pairwise comparison revealed that, although many IK G_1_/G_0_ cells remained neighbors, 30% of cells switched their partners; in contrast, most S/G_2_/M pairs remained neighbors (Fig. 5E). Pharmacological inhibition of actomyosin-based motility with the inhibitor blebbistatin repressed IK G_1_/G_0_ condensation and abrogated progression of tooth development (Fig. 5F). These findings together confirmed active migration of individual IK cells.

### Dynamics between Wnt^Hi^ and *Shh* cell populations regulate the maturation and maintenance of the IK

**Fig. 6.**
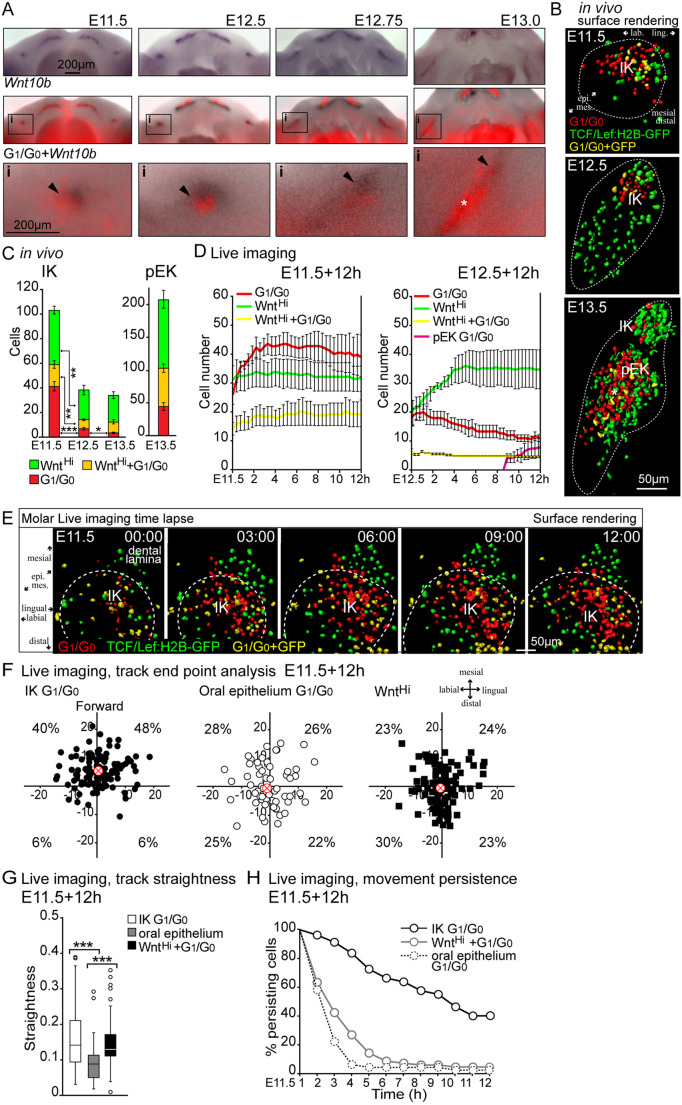
**Dynamics between Wnt^Hi^ and *Shh* cell populations regulates IK maturation and maintenance.** (A) Fucci G_1_/G_0_ fluorescence images overlaid with whole-mount DIG *in situ* hybridization for *Wnt10b.* IK is indicated by arrowheads and emerging pEK by an asterisk. i indicates the area shown at higher magnification underneath. (B) High-resolution analysis of sagittal optical sections of fixed samples of Fucci G_1_/G_0_ (red) and TCF/Lef:H2B-GFP (green) patterns. The tooth epithelium perimeter is indicated by white-dotted lines. At E11.5, TCF/Lef:H2B-GFP high intensity (Wnt^Hi^, visualized with nuclear surface rendering) was present in the dental lamina and the molar IK G_1_/G_0_ condensate was located next to a border of Wnt^Hi^ cells. At E12.5, IK G_1_/G_0_-only cells were surrounded by Wnt^Hi^ cells. At E13.5, G_1_/G_0_ and Wnt^Hi^ cells were present in the presumptive pEK region (asterisk). (C) Quantification of cells in Wnt^Hi^, G_1_/G_0_ and double-positive cell populations in fixed samples (data are mean±s.e.m., IK *n*_E11.5_=413; *n*_E12.5_=192; E13.5 *n*_IK_=136, *n*_pEK_=831, non-parametric Student's *t*-test, *P≤0.05, **P≤0.01, ***P≤0.001). (D) Quantification of cell number in Wnt^Hi^, G_1_/G_0_ and double-positive cell populations from live tissue imaging at E11.5+12 h and E12.5+12 h (data are mean±s.e.m. *n*_E11.5+12h_=*n*_E12.5+12h_=3). (E) Surface-rendering still images of E11.5+12 h molar time-lapse, Fucci G_1_/G_0_, TCF/Lef:H2B-GFP and double-positive cells. At E11.5+3 h onwards, the G_1_/G_0_ cell population started differentiating closely juxtaposed to Wnt^Hi^ cells, with an increasing number of cells transitioning to G_1_/G_0_. (F) Track end point analysis (median is indicated by a red cross, *n*_IK G1/G0_=128, *n*_oral G1/G0_=63, *n*_WntHi_=104). Molar IK G_1_/G_0_ cells showed preferential distribution toward the dental lamina Wnt^Hi^ region (forward). (G) Track straightness in molar IK G_1_/G_0_, oral epithelial G_1_/G_0_ and TCF/Lef:H2B-GFP+ cell populations (data are mean±s.e.m., *n*_IK G1/G0_=128, *n*_oral G1/G0_=42, *n*_WntHi_ =67, Mann–Whitney *U*-test, ***P≤0.001). (H) Decay in cell movement persistence (plots represent minimum, 25th percentile, median, 75th percentile and maximum values; *n*_IK G1/G0_=80, *n*_oral G1/G0_=50, *n*_WntHi_=71).

Our live imaging analysis suggested that TCF/Lef:H2B-GFP reporter-expressing Wnt^Hi^ cells were closely juxtaposed to *Shh*-expressing G_1_/G_0_ IK cells but appeared to comprise two different cell populations that remained in close contact with each other throughout bud development (Fig. 6, Movies 4 and 6, Fig. S1F). The Shh pathway is an important modulator of Wnt signaling during several stages of tooth development. Studies in mouse mutants implied that Shh is a downstream target of Wnts and also an inhibitor of Wnt signaling via a negative-feedback loop (Sarkar et al., 2000; Sarkar and Sharpe, 1999). Thus, we next investigated the behavioral dynamics and the molecular identity of the two cell populations.

*In situ* hybridization analysis of Fucci specimens revealed that the IK G_1_/G_0_ cells colocalized with *Shh* (Fig. S1A). Expression in the placode of a canonical Wnt, *Wnt10b*, has previously been reported (Liu et al., 2008). Hybridization of *Wnt10b* in the Fucci G_1_/G_0_ reporter at E11.5 revealed that *Wnt10b* expression was detected, partially overlapping, but predominantly anterior to, the G_1_/G_0_ focus (Fig. 6A). By E12.5, the dense IK G_1_/G_0_ colocalized with the *Shh* signal, whereas *Wnt10b* expression covered a larger area surrounding the IK G_1_/G_0_ condensate. By E12.75, the diffuse *Wnt10b* expression continued to reside in a wider area in the molar mesiolingual tip. At E13.0, the G_1_/G_0_ area was barely discernible and *Shh* and *Wnt10b* were downregulated. Concomitantly, G_1_/G_0_, *Shh* and *Wnt10b* expression appeared in the emerging pEK (Fig. 6A, Fig. S1A). These data show that there was a complete spatial correlation with *Shh* and G_1_/G_0_ signal throughout early molar morphogenesis, but that *Wnt10b* expression was also seen in the area juxtaposing the G_1_/G_0_ focus anteriorly.

High-resolution analysis and live imaging of G_1_/G_0_ and TCF/Lef:H2B-GFP patterns in fixed samples showed that the *Shh*-G_1_/G_0_ cell population initiated at E11.5 was closely juxtaposed to Wnt^Hi^ cells (Fig. 6B, Movie 4). By E12.5, Wnt^Hi^ cells surrounded the *Shh*-G_1_/G_0_ cells and there was little distribution of TCF/Lef:H2B-GFP+ cells in the growing bud prior to pEK appearance (Fig. 6B, Movie 6). At E13.5, Wnt^Hi^ cells were present in the pEK, with G_1_/G_0_ cells distributed more centrally (Fig. 6B). Quantification of *Shh*-G_1_/G_0_ and Wnt^Hi^ cell populations in fixed samples showed a decrease in *Shh*-G_1_/G_0_ cell number at E12.5, concomitant with TCF/Lef:H2B-GFP downregulation and appearance of apoptosis specifically in the IK cells (Fig. 6C, Fig. S5A,B). This was consistent with the hypothesis that canonical Wnt signaling activity and *Shh* expression participate in the maintenance of the IK. To examine this dynamic more closely, we quantified the cell *Shh*-G_1_/G_0_ and Wnt^Hi^ populations with live imaging in E11.5 and E12.5+12 h molars. Quantification at E11.5+12 h showed that the number of Wnt^Hi^ cells remained stable for the 12 h follow-up; in contrast, the *Shh*-G_1_/G_0_ cell population increased by 1.5-fold (Fig. 6D). Analysis of E12.5+12 h cells showed an increase in Wnt^Hi^ cells throughout the bud, reaching a plateau after 4 h; IK *Shh*-G_1_/G_0_ cells showed a constant decrease and G_1_/G_0_ cells appeared in the pEK from 9 h onward (Fig. 6D).

Analysis of individual cell populations contributing to the initiation of the molar placode showed a border region with an accumulation of Wnt^Hi^-*Wnt10b* cells in the dental lamina and G_1_/G_0_-*Shh* cells starting to differentiate closely juxtaposed to this region (Fig. 6E). Analysis of cell movement showed differential patterns in the Wnt^Hi^-*Wnt10b* and G_1_/G_0_-*Shh* cell populations: track end-point analysis showed specific preferential movement of G_1_/G_0_-*Shh* IK cells toward the dental lamina Wnt^Hi^ cells (Fig. 6F) with high track straightness (Fig. 6G) and high directional persistence in the G_1_/G_0_-S*hh* IK cells compared with oral epithelial G_1_/G_0_ and dental lamina Wnt^Hi^ cells (Fig. 6H).

The differential distribution and cellular behaviors of Wnt^Hi^ and *Shh*-G_1_/G_0_ cells in the molar signaling centers suggest that they act in concert to initiate signaling center cell differentiation in the very early stages of tooth formation. The boundary between the two cell populations defines the position of the emerging molar IK and orients the directional migration pattern for condensation. Furthermore, decreased Wnt signaling resulted in *Shh* downregulation and IK clearance.

### Modulation of canonical Wnt signaling affects IK cell dynamics and tooth bud shape

The cell movement data suggested the presence of a chemotactic gradient from the dental lamina Wnt^Hi^-*Wnt10b* cells directing the movement and/or condensation of the G_1_/G_0_-*Shh* cell population in molars. *Wnt10b* has been previously implicated as a paracrine chemotactic factor in epithelial cancer contexts (Aprelikova et al., 2013; Chen et al., 2017). To explore whether this dynamic occurs in developing molars, we modulated canonical Wnt signaling levels by stimulation with Wnt3a and by inhibition with a Wnt antagonist, XAV939, which acts by stimulating β-catenin degradation and stabilization of axin. E11.5 explants were treated with Wnt3a/XAV939 in the growth medium for 24 h. Alternatively, a recombinant Wnt10b-soaked/control bead was placed next to the placode at E11.5 and the explants were followed by imaging for up to 16 h to ensure good tissue health.

**Fig. 7.**
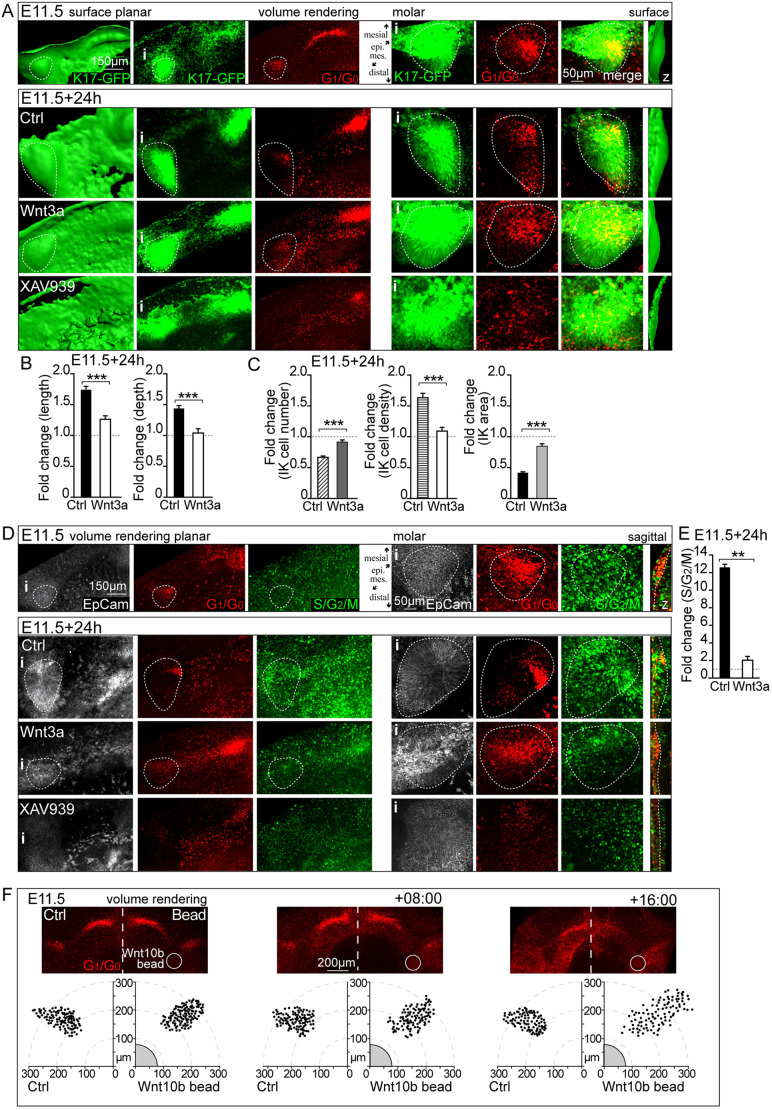
**Modulation of canonical Wnt signaling affects IK cell dynamics and molar bud shape.** (A) Confocal fluorescence images of explant cultures showing K17-GFP (epithelium, green), Fucci nuclei G_1_/G_0_ (red) and the tooth placode/bud epithelium perimeter (dotted line). i indicates the area shown at higher magnification on the right and z the surface rendering side view. Canonical Wnt signaling levels were modulated from placode stage at E11.5+24 h by either stimulation with Wnt3a or inhibition with XAV939. Stimulation resulted in a flat bud with persistant G_1_/G_0_ IK cells throughout the invagination. Inhibition led to complete loss of the G_1_/G_0_ condensate and abrogated invagination. (B) Quantification of bud dimensions in Wnt3a-stimulated and control cultures (data show the fold change over E11.5 and are mean±s.e.m.; *n*_ctrl_=12, *n*_Wnt3a_=10, Mann–Whitney *U*-test, ***P≤0.001). (C) Quantification of IK cell number, density and area in Wnt3a-stimulated and control cultures (data show the fold change over E11.5 and are mean±s.e.m., *n*_ctrl_=12, *n*_Wnt3a_=10, Mann–Whitney *U*-test, ***P≤0.001). (D) Stimulation with Wnt3a in Fucci G_1_/G_0_ (red) and S/G_2_/M (green) cultures resulted in lack of IK condensation followed by a drastic loss of cell proliferation in the bud. Inhibition with XAV939 resulted in the loss of the G_1_/G_0_ IK condensate and absence of proliferation and invagination. i indicates the area shown at higher magnification on the right and z the surface rendering side view. (E) Quantification of cell proliferation in Wnt3a-stimulated and control cultures (data show the fold change over E11.5 and are mean±s.e.m.; *n*_ctrl_=7, *n*_Wnt3a_=8, Mann–Whitney *U*-test, **P≤0.01). (F) A Wnt10b recombinant protein-releasing bead was placed next to the IK (visualized with a Fucci G_1_/G_0_ reporter) distally to the placode. Explants were imaged at E11.5, after 8 h and after 16 h. Morphogenesis and IK condensation proceeded normally in control buds without beads. Application of the Wnt10b-releasing bead resulted in a loss of condensation of G_1_/G_0_ IK cells. Instead, the G_1_/G_0_ IK cells were spread out toward the Wnt10b bead.

We used K17-GFP to visualize the shape of the epithelium and Fucci G_1_/G_0_ for IK cell distribution in the developing placode/bud. The epithelial placode grows mostly in depth at this time and stimulation with Wnt3a resulted in a flat bud compared with control (Fig. 7A,B), with a consistent number of G_1_/G_0_ IK cells spread out throughout the invagination (Fig. 7A,C). Inhibition of active Wnt signaling with XAV939 resulted in a complete loss of G_1_/G_0_ condensate together with a loss of invagination (Fig. 7A). To study whether the lack of IK condensation and the loss of invagination, with Wnt modulation, was caused by a lack of bud cell proliferation, we treated Fucci G_1_/G_0_; S/G_2_/M mandibles with either Wnt3a or XAV939. Stimulation with Wnt3a resulted in a lack of IK condensation, followed by a drastic loss of cell proliferation in the bud (Fig. 7D,E). Inhibition with XAV939 resulted in the loss of the G_1_/G_0_ IK condensate and absence of proliferation and invagination (Fig. 7D).

To dissect the role of *Wnt10b* in IK condensation, we placed a Wnt10b-releasing or control bead close to the IK on the lingual side of the placode, according to a previously well-established protocol (Dassule and McMahon, 1998); the explants were imaged at E11.5 +8 h and +16 h. Morphogenesis and IK condensation proceeded normally in both the control bud and the bud with the control bead (Fig. 7F, Fig. S6A). In contrast, the bud with the Wnt10b bead showed a loss in condensation of the G_1_/G_0_ IK cells (Fig. 7F). Instead, the G_1_/G_0_ IK cells were spread out toward the Wnt10b bead. In the observed time frame, placode growth occurs mostly in the mesial to distal axis. Accordingly, Wnt10b bead-treated buds showed a decrease in elongation compared with control, together with a decrease in G_1_/G_0_ IK cell density (Fig. S6B). The changes in IK cell distribution and bud shape were also accompanied by a decrease in cell proliferation (Fig. S6C,D).

## DISCUSSION

The reiterative genetic regulation of tooth development via signaling centers is conserved across tooth types, but less understood is how it is interpreted into different cellular behaviors to regulate tooth shape and size. In the present study, we identified a molar IK signaling center that is necessary for the progression of tooth development in the early stages of mammalian tooth morphogenesis. We show with live imaging, 4D whole-mount analyses, and functional ablation studies that the IK arises in the placode and is a functional signaling center that drives proliferative growth prior to the successive EKs. Molar IK cell dynamics displays the hallmarks of ectodermal signaling centers: cell cycle exit and condensation, and silencing through apoptosis. Cell cycle exit coupled to active condensation takes place not only in teeth, as shown here and by Ahtiainen et al. (2016), but also in hair placodes (Ahtiainen et al., 2014). Condensation of the IK via active cell movement is necessary for progression of tooth budding, because inhibition of actomyosin contractility and modulation of condensation guiding Wnt signaling levels compromised the function of the signaling center. Cell condensation could be a universal mechanism to trigger both ectodermal signaling center differentiation and cell cycle exit by contact inhibition of cell proliferation, eventually regulating the timing of signaling center silencing by initiating mechanical crowding-induced apoptosis.

Tissue recombination studies have shown that the instructive potential in the tooth first resides in the epithelium and shifts only later to the mesenchyme (Lumsden, 1988; Mina and Kollar, 1987). EKs require inductive signals from the mesenchyme, but it is plausible that the IK-inducing signal comes from planar epithelial signaling. Wnt7b, Wnt3 and Shh already have mutually exclusive expression in the presumptive oral and dental ectoderm at E10.5 (Sarkar and Sharpe, 1999; Sarkar et al., 2000), and it appears that different Wnt expression patterns and *Shh* determine the ectodermal boundaries of competence at a very early stage. *Shh* is possibly a downstream target of Wnts and also a negative-feedback inhibitor. Spatial inhibition of *Wnt10b* by *Shh* has been reported in teeth: Shh-coated beads repressed *Wnt10b* but no other epithelial markers (Dassule and McMahon, 1998). Constitutive activation of epithelial Wnt/β-catenin, somewhat later than E12.5, induced multiple patches of signaling center markers, including *Shh* and *Wnt10b* at E13-E14, and ectopic teeth (Jarvinen et al., 2006; Liu et al., 2008). Our work shows that *Wnt10b* and *Shh* are differentially expressed during molar initiation and that these cell populations remain functionally separate. However, close interaction between the G_1_/G_0_-*Shh* and Wnt^Hi^-*Wnt10b* expressing cells is crucial in the positioning and maintenance of the molar IK. Wnt10b has been implicated as a paracrine chemotactic factor in cancers (Aprelikova et al., 2013; Chen et al., 2017). The migration of G_1_/G_0_-*Shh* IK cells toward the canonical Wnt gradient and specific area of endogenous *Wnt10b* expression, and the distribution of IK cells toward the exogenous source of recombinant Wnt10b suggest that *Wnt10b* carries an instructive role in signaling center condensation.

We show that molar invagination and growth take place through cell proliferation in both basal and suprabasal bud cell populations, driven by the non-proliferative IK. Shh has been proposed a primary inducer of proliferation in some experimental settings, whereas other studies suggest a role in bud cell rearrangement (Hardcastle et al., 1998; Cobourne et al., 2009; Prochazka et al., 2015; Li et al., 2016b). *Shh* expression is a hallmark of signaling centers and, although autocrine signaling cannot be ruled out, most of the responsive cells appear to reside elsewhere: at later stages, the pEK expresses *Shh*, whereas the receptor *Ptch* and downstream targets *Gli1/2/3* are expressed in the mesenchyme (Vaahtokari et al., 1996a; Hardcastle et al., 1998). During initiation of invagination, the expression of both *Ptch1* and *Gli1/2* have been reported in the emerging epithelial bud at E12.0 (Dassule and McMahon, 1998; Hardcastle et al., 1998). Notably, in our analyses, proliferation coincided with this. In agreement, early findings from conditional *Shh* mutants showed smaller teeth and posteriorly misplaced buds (Dassule and McMahon, 1998; Dassule et al., 2000).

Shh has been shown to be protective of early apoptosis in the tooth (Cobourne et al., 2001). Apoptosis is a mechanism used to silence signaling centers in teeth and in the apical ectodermal ridge of the limb, as well as during embryonic brain development (Vaahtokari et al., 1996b; Matalova et al., 2004; Nonomura et al., 2013). The interplay between Wnt^Hi^ and *Shh*^+^ cells might serve as a feedback mechanism regulating the timing of apoptosis in the IK.

Contrary to the classical view of tooth development, the vestigial tooth hypothesis suggests a presence of transient vestigial tooth buds anterior to the mandibular first molar (M1), termed MS and R2 (Hovorakova et al., 2011, 2013; Prochazka et al., 2010; Peterkova et al., 2014). These studies rely on 3D reconstructions from histological sections, whereas evidence of initiating and regressing buds comes from an observation of temporally distinct expression domains of signaling center marker patterns: at each stage, each domain is detected in a given position moving anterior to posterior as the mandible grows. With new live tissue-imaging methods and lineage-tracing approaches, direct spatiotemporal evidence of the relationship of these structures is now available. Studies in the mouse mandible using mouse mutants, fluorescent reporters and live imaging (Prochazka et al., 2015; Ahtiainen et al., 2016) describe the relationship between these expression domains directly in living tissue. Prochazka et al. (2015) explored the mechanisms of M1 initiation and positioning, reporting intraepithelial migration toward the Shh-expressing placode. This study reported no evidence of initiating/regressing buds, but rather suggested the contribution of a cell population, originating from the jaw hinge, to the M1. Ahtiainen et al. (2016) identified an IK in the incisor and showed the IK and EK to be clonally distinct parts of the same incisor bud, a finding later confirmed by a conventional lineage-tracing approach (Du et al., 2017). A study in molar sections from placode to bud stage did not support the hypothesis of bud regression (Li et al., 2016b). This body of evidence, together with the current study, shows that the IK arising in the molar placode corresponds to MS; instead of regressing, this structure drives tooth growth as an integral part of the bud, maturing into R2. The IK is removed apoptotically and does not contribute to the pEK in M1, as previously suggested for R2.

In normal development in mice, tooth formation in the diastema region is inhibited. However, there are mouse mutants that have an extra tooth in front of M1 or continuous induction of signaling centers and supernumerary teeth in the oral cavity. Mouse mutants of Sprouty family members, negative-feedback regulators of Fgf signaling, exhibit in a low frequency a small supernumerary tooth in front of the M1 (Klein et al., 2006). Similarly, mouse mutants constitutively overexpressing Ectodysplasin (Eda) in the epithelium (K14-Eda) occasionally have an extra tooth in this position (Mustonen et al., 2003). Eda regulates several signaling center genes (including *Wnt10a*, *Wnt10b*, *Fgf20* and *Shh*) and correct levels of Fgf signaling are necessary to induce and sustain signaling center and tooth development (Haara et al., 2009; Prochazka et al., 2015; Pummila et al., 2007; Voutilainen et al., 2012; Yu and Klein, 2020). Eda is further regulated by Wnt/β-catenin signaling during early molar morphogenesis, and Wnt signaling is required for early tooth morphogenesis, as shown by the current study and Andl et al. (2002). Wnt hyperactivation through Lef1 or overexpression of constitutive active of β-catenin results in large numbers of supernumerary teeth in adult animals (Nakamura et al., 2008; Jarvinen et al., 2006). In light of these mouse mutant phenotypes, it is plausible that supernumerary teeth could arise from the disturbed lifecycle (from initiation and maturation to silencing via apoptosis) of the IK signaling centers. In the current study, Wnt hyperstimulation resulted in an expanded G_0_/G_1_ domain, although the time window of the experiments did not allow direct inference of whether extended Wnt hyperstimulation exposure could support an accessory group of cells forming a supernumerary IK. Wnt inhibition blocked both prospective IK cells from entering G_0_/G_1_ and abrogation of condensation, leading to a complete loss of epithelial invagination. This is reminiscent of the Lef1-knockout mouse with arrested tooth development at the bud stage (van Genderen et al., 1994). However, many of the Cre-driver lines that have commonly been used in tooth research become fully active only after the appearance of the dental placode. The signaling requirements between different tissue compartments may be different at later stages. This could explain the effects of canonical Wnt hyperstimulation on initiation stage molars in this study, which are reminiscent of effects seen in hair follicle placode initiation (Ahtiainen et al., 2014), and possible discrepancies between later-stage Wnt hyperstimulation models.

We demonstrate that the pEK in the molar is formed *de novo* without clonal contribution from the IK and that the IK is silenced apoptotically upon pEK appearance. This differs mechanistically from signaling centers later in molar development, where the pEK contributes cells to sEKs (Du et al., 2017). Interestingly, the presence of a functional IK is a prerequisite for molar bud growth. We also demonstrate that removing the IK arrests tooth development and results in a loss of pEK formation. The size of the pEK also appears to be dependent on the IK, either directly or indirectly through regulation of the epithelial bud size. However, the development of teeth is conserved, being driven by the iterative use of signaling centers. In addition, we show functionally that progression of early molar morphogenesis is dependent on the IK signaling center, which arises in the placode and exhibits hallmarks of ectodermal signaling centers. This study is the culmination of a body of functional evidence strongly supporting the classical view of tooth development, which proposes that each placode gives rise to a respective functional tooth. What differentiates the IK from the later signaling centers at a transcriptomic level will be of particular interest for future studies.

## MATERIALS AND METHODS

### Animals, tissues preparation and culture treatments

All mouse studies were approved by the National Animal Experiment Board. Transgenic mouse reporter lines were as follows: fluorescent cell cycle indicator (Fucci) mice express a nuclear red fluorescent reporter in G_1_/G_0_ phase (Cdt1-mKO) and a green fluorescent reporter in S/G_2_/M phases (Gem-mAZ) (Sakaue-Sawano et al., 2008); Shh^GFPCre^ mice (005622, Jackson Laboratories) express GFP consistent with endogenous *Shh* loci, thus visualizing the signaling centers (Harfe et al., 2004); K17-GFP mice (023965, Jackson Laboratories) visualize the tooth epithelium; TCF/Lef:H2B-GFP mice (013752, Jackson Laboratories) are indicators of Wnt/β-catenin signaling, containing several copies of TCF/Lef1 DNA-binding sites driving expression of the H2B-EGFP fusion protein; and FGF20^βGal^ mice have an Fgf20–β-galactosidase (βGal) knock-in allele (Huh et al., 2012). Embryos were staged according to limb morphological criteria; vaginal plug day was E0.5 (Martin, 1990).

Embryonic mandibles were dissected at E11.5-E13.5 and whole-mount explants were fixed from 2 h to overnight in 4% paraformaldehyde (PFA) or cultured in a Trowell-type tissue culture as described previously (Narhi and Thesleff, 2010). For live-imaging experiments, tissues were maintained in Dulbecco's Modified Eagle Medium/Nutrient Mixture F-12 (DMEM/F12) without Phenol Red and supplemented with 50 U/ml penicillin, 50 µg/ml streptomycin, 10% fetal calf serum (FCS) and HEPES 15 mM (Gibco). For inhibitor/activator treatments, samples were dissected at E11.5 or E11.75; the growth medium was then treated for 24 h by adding: (1) vehicle; (2) blebbistatin to inhibit actomyosin-mediated cell motility (100 µM; Sigma-Aldrich); (3) recombinant Wnt3a (10 ng/ml; R&D Systems) for stimulation or (4) XAV939 (10 μM; Tocris) to inhibit canonical Wnt signaling. For bead implantation, beads were treated similarly as reported previously (Dassule and McMahon, 1998). Briefly, heparin acrylic beads (MCLAB) were incubated with 100 μg/ml recombinant human Wnt10b protein (0.1 mg/ml; R&D Systems) at 37°C for 30 min. Control beads were soaked with similar concentrations of bovine serum albumin (BSA) under the same conditions. Protein-soaked beads were stored at 4°C and used within 1 week. Beads were applied on tissue explant cultures at E11.5 distally of the placode and tissues were imaged at the start point, after 8 and 16 h to ensure good tissue health.

### Ethics approval

All mouse studies were approved and carried out in accordance with the guidelines of the Finnish National Animal Experimentation Board under licenses KEK15-027, KEK18-028, ESAVI/1284-04.10.07/2016 and ESAVI/19567/2019.

### Whole-mount immunofluorescence, fluorescence microscopy and *in situ* hybridization

For whole-mount immunofluorescence staining, fixed tissues were permeabilized with 0.5% TritonX-100 for 2 h at room temperature (RT) and washed with PBS. Unspecific staining was blocked by incubation in 5% normal donkey/goat serum, 0.3% BSA, 0.1% Triton X-100 in PBS for 1 h at RT. Tissues were incubated overnight at 4°C with the primary antibody rat polyclonal anti-mouse CD326 (EpCam, 1:1000; Pharmingen, 552370), rabbit polyclonal βGal (1:400; MP Biomedicals, 0855976) or rabbit polyclonal cleaved caspase 3 (1:400; Cell Signaling Technology, 9664) and detected with Alexa Fluor-488 or Alexa Fluor-647-conjugated secondary antibodies (1:500, Thermo Fisher, A48265, A32731, A32733); nuclei were stained with Hoechst 33342. Tissues were mounted with Vectashield (Vector Laboratories) and imaged with either a Leica Biosystems TCS SP5 microscope and HC PL APO 10×/0.4 (air), HCX PL APO 20×/0.7 Imm Corr (water, glycerol, oil) Lbd.bl and HCX APO 63×/1.30 Corr (glycerol) CS 21 objectives or with a Zeiss LSM700 microscope and HC PL APO 10×/0.45 (air) and LD LCI PL APO 25×/0.8 Imm Corr (water, glycerol, oil) objectives. For analysis of TCF/Lef:H2B-GFP signal intensities, the cut-off values for high and low-expressing cells were adjusted according to overall signal intensity in each sample. All results represented at least three independent experiments.

For combined fluorescence and whole-mount *in situ* hybridization analyses, fluorescent imaging of fixed Fucci G_1_/G_0_ reporter whole-mount mandibles was carried out first with a Zeiss SteREO Lumar.V12 microscope, NeoLumar S 0.8×/WD 80 mm objective and Zeiss Axiocam MRm3 CCD camera. The samples were then subjected to whole-mount *in situ* hybridization with DIG-labeled probes specific for *Shh* or *Wnt10b*, performed as described previously (Wang and Shackleford, 1996; Fliniaux et al., 2008; Shirokova et al., 2013). Imaging of the hybridization signal was peformed with the same Zeiss Lumar microscope and Zeiss AxioCam ICc1 CCD camera, and the images were transposed.

### Fluorescence confocal microscopy, time-lapse imaging and laser ablation

For 3D time-lapse imaging, dissected tissues were allowed to recover for a minimum of 2 h prior to imaging. The explants were imaged as described previously (Ahtiainen et al., 2014, 2016) using an upright Leica Biosystems TCS SP5 microscope with a HC PL APO 10×/0.4 (air) objective in a Trowel-type culture setup. *Z*-stacks of 3 µm optical sections were acquired at 20 min intervals. Good tissue health was confirmed by a lack of pyknotic nuclei and frequency of mitoses in every acquired *z*-stack. For determination of cell cycle status and cell quantification, only cells that were distinctly identified as either G_1_/G_0_ or S/G_2_/M were scored. For Wnt/β-catenin signaling activity, TCF/Lef:H2B-GFP cells were scored individually for median fluorescence intensity in each nucleus and for presence of G_1_/G_0_ signal. All results represented at least three independent experiments.

Laser ablations were performed with an upright Leica Biosystems TCS SP5 microscope, HC PL APO 10×/0.4 (air) objective and a tunable Ti:Sapphire pulsed infrared (IR) laser (Spectra Physics, MaiTai, tunable range 690-040 nm) at RT with an 800 nm excitation wavelength and 2.95 W laser power (100%) for 3-10 s. The pulse was targeted to either the IK or control area and visualized with the Fucci reporter using a 20-40× zoom factor. Efficiency and specificity of ablation was verified by acquiring confocal fluorescence *z*-stacks of the sample immediately after ablation. After 24 h of culture, tissues were fixed in 4% PFA for 2 h at RT or overnight at 4°C, Fucci cell cycle reporter samples were immunofluorescence stained with EpCam to visualize the epithelium; all samples were stained with Hoechst 33342 to visualize nuclei. Samples were imaged with a Zeiss LSM700 microscope, with HC PL APO 10×/0.45 (air) and LD LCI PL APO 25×/0.8 Imm Corr (glycerol) objectives. Specificity of ablation was verified by the absence of Fucci G_1_/G_0_ phase (Cdt1-mKO)-positive cells in the IK region and ablation of only the epithelial compartment visualized with the K17-GFP reporter and Hoechst staining. Good tissue health of the directly adjacent, off-target epithelial and mesenchymal tissues was confirmed by a lack of pyknotic nuclei and presence of normal physiological cell proliferation patterns of the Fucci reporter.

### Visualization, quantitative and statistical analyses of experimental data

Analyses of images and quantitative measurements were performed with Imaris 9.0.1 (Bitplane) and ImageJ software. Images were processed for presentation with Photoshop CC and Illustrator CC software (Adobe Systems). Statistical analysis and further graphing were performed with PAST (http://folk.uio.no/ohammer/past/; Hammer et al., 2001) and SPSS Statistics (IBM) software.

All measurements were taken from whole-mount volume renderings of confocal optical *z*-stacks. Definition of the tooth bud epithelium perimeter in each experiment was obtained from stereoscopic 3D renderings in the Imaris software. The epithelium was visualized with either the transgenic K17-GFP reporter or EpCam staining (Fig. S7), both specific to the tooth epithelium. In addition, when nuclei were visualized with a fluorescent label or reporter (Hoechst or Fucci, respectively), the border between the epithelium and mesenchyme was readily visible in optical sections of whole-mount samples because of different cellular organization in the mesenchyme and epithelium (Fig. S7). The perimeter of the epithelial thickening was defined from optical *z*-sections in multiple orientations and from whole-mount volume rendering observed from multiple angles. Surface rendering was carried out using Imaris software by thresholding based on the epithelial fluorescence signal. All surface renderings show the epithelium presented from the mesenchymal side toward the epithelium (Fig. S7). Volume renderings represent the whole tissue volume from the confocal *z*-stack and are presented from the mesenchymal side toward the epithelium. In some surface renderings, only the epithelial compartment is shown, and this is stated in the relevant figure legends.

To quantify cell density, individual cell borders were visualized and traced in 3D with EpCam staining of whole-mount tissues. Cell densities were quantified by masking a volume in tooth epithelium or an equal volume in the oral epithelium, and were defined as the area occupied by the cell (selecting a cross-section in 3D view in the middle of the cell). Box-and-whiskers plots represent minimum, 25th percentile, median, 75th percentile and maximum values for each dataset. Differences between groups were assessed with the Mann–Whitney *U*-test or non-parametric Student's *t*-test.

All cell movement, follow-up and division analyses were performed using stereoscopic 3D renderings, allowing exact localization of cells in both 3D and time (Ahtiainen et al., 2014, 2016). Individual cell track length and net displacement were measured in signaling center and oral epithelial cells. Track plots and cell movement analyses present all marker-positive cells detected in the tooth/placode or respective oral epithelium, unless otherwise stated. The distribution of cell trajectory displacement angles was analyzed with the Rayleigh test (H_0_=random, *P*>0.05). For IK G_1_/G_0_ pairwise cell trajectory analysis, tissues were live imaged at E11.5+12 h. G_1_/G_0_ cells were divided into pairs in their original position [within close proximity (≤15 µm) of each other], traced to the end position and the centroid distance was then measured. For track end-point analysis, the starting point of each track was placed at the origin and track displacement at the end point was plotted. For the analysis of the decay of cellular persistence in directional migration, we first determined the angle of cell migration during the first hour of observation for the initial orientation of the cells. At each following time point, cells that had not yet turned more than 90° from their starting angle were considered directionally persistent.

## Supplementary Material

Supplementary information
