# Supplementary material for: The initiation knot is a signaling center required for molar tooth development
Source: Development. 2021 Apr 29;148(9):dev194597. doi: 10.1242/dev.194597 (PMC8126415; doi:10.1242/dev.194597)
Supplement: Supplementary information [file develop-148-194597-s1.pdf]

Supplemental Figure S1. (related to Figure 1)

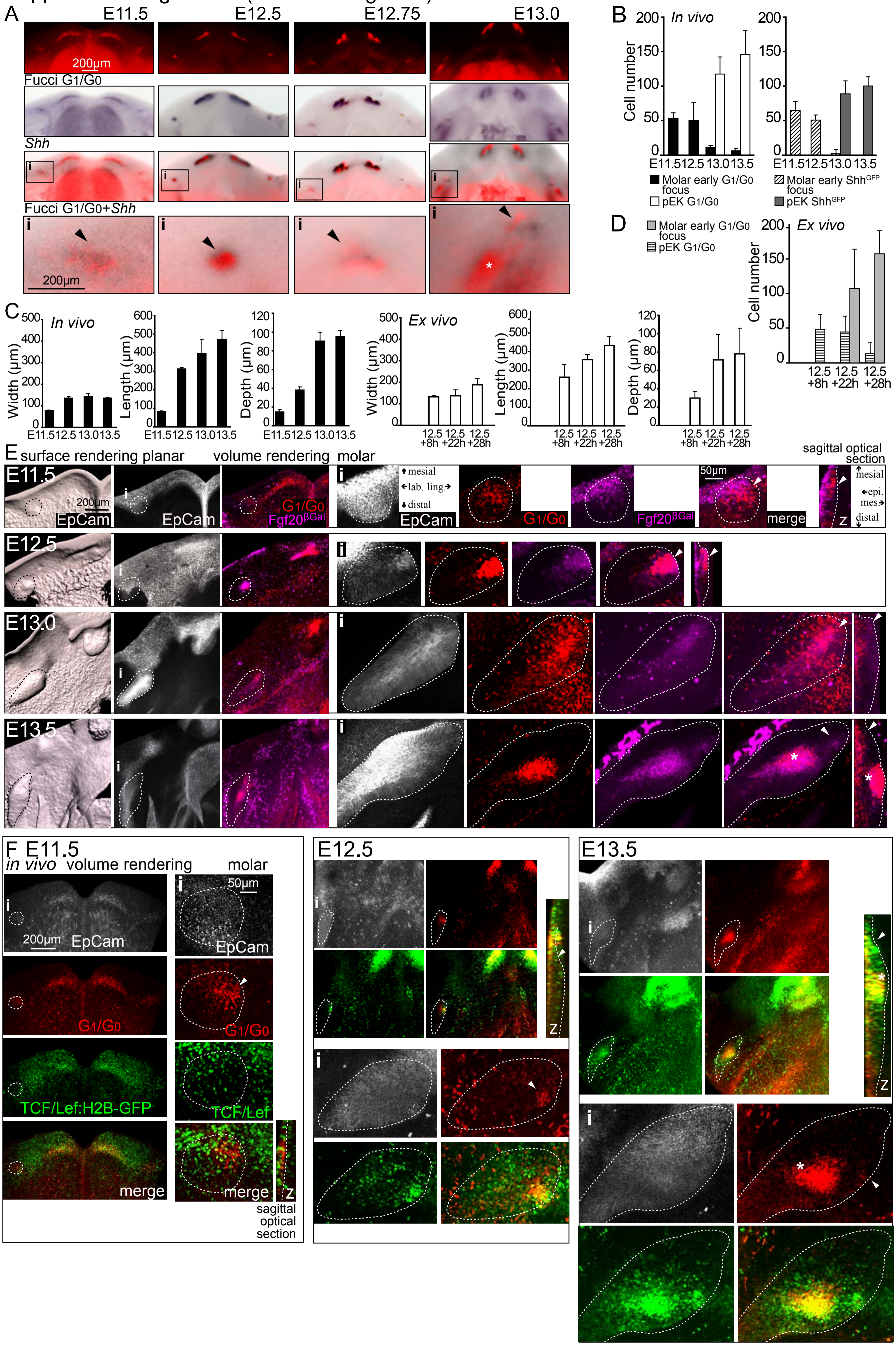

Development • Supplementary information

Related to Figure 1.

Figure S1. Mandible explants grown *in vitro* show same cell behavioral and morphogenetic developmental patterns as in *in vivo*

For functional live tissue imaging analyses we first verified that cultured explants developed, in respect to G_1/G_0 cell population dynamics and bud growth, comparably to *in vivo* development. (A) Fucci G_1/G_0 fluorescence images overlaid with whole-mount DIG *in situ* hybridization with a probe specific for *Shh*. Molar IK G_1/G_0 focus (arrowhead), emerging pEK (asterisk). (B) Quantifications of G_1/G_0 and Shh-GFP cells in the developing molar placode and bud *in vivo* (G_1/G_0 embryos $N_{E11.5}$=7, $N_{E12.5}$=11, $N_{E13.0}$=4 $N_{E13.5}$=5, error bars±SEM)(Shh^{GFP} embryos $N_{E11.5}$=4, $N_{E12.5}$=11, $N_{E13.0}$=3 $N_{E13.5}$=5, error bars±SEM). (C) Quantification of molar bud dimensions *in vivo* (black bars) and *ex vivo* cultured explants (white bars) Dimensions in width and length were similar *in vivo* and cultured specimens with somewhat flatter bud after culturing ($N_{E11.5}$=5, $N_{E12.5}$=11, $N_{E13.0}$=4, $N_{E13.5}$=5, $N_{E12.5+8h}$=4, $N_{E12.5+22h}$=10, $N_{E12.5+28h}$=7, mean±SD). Explants show a similar pattern of morphogenesis to *in vivo* with a slightly flatter bud shape. (D) Quantification of G_1/G_0 cells in the developing molar placode and bud *in vitro* in cultured explants (embryos $N_{E12.5+8h}$=2, $N_{E12.5+22h}$=8, $N_{E12.5+28h}$=7, mean±SEM) There was a slight overall increase in G_1/G_0 cell observed in culture conditions compared to *in vivo*. (E) Fgf20$^{\beta Gal}$ reporter βGal immunofluorescence staining (magenta), Fucci G_1/G_0 (red), epithelium (EpCam, grey), tooth epithelium perimeter (dotted line), sagittal optical section (z), arrowhead (the early focus corresponding to the initiation knot, IK), asterisk (prospective pEK). The Fgf20$^{\beta Gal}$ signaling center marker showed expression corresponding to G_1/G_0 foci throughout placode and bud morphogenesis and in the emerging pEK. (F) Another signaling center marker, the fluorescent canonical Wnt signaling reporter TCF/Lef:H2B-GFP (green), overlapped with the Fucci G_1/G_0 foci; however, a proportion of the G_1/G_0 cells (red) remained distinct, not showing TCF/Lef:H2B-GFP reporter activity.

Development • Supplementary information

Supplemental Figure S2. (related to Figure 2)

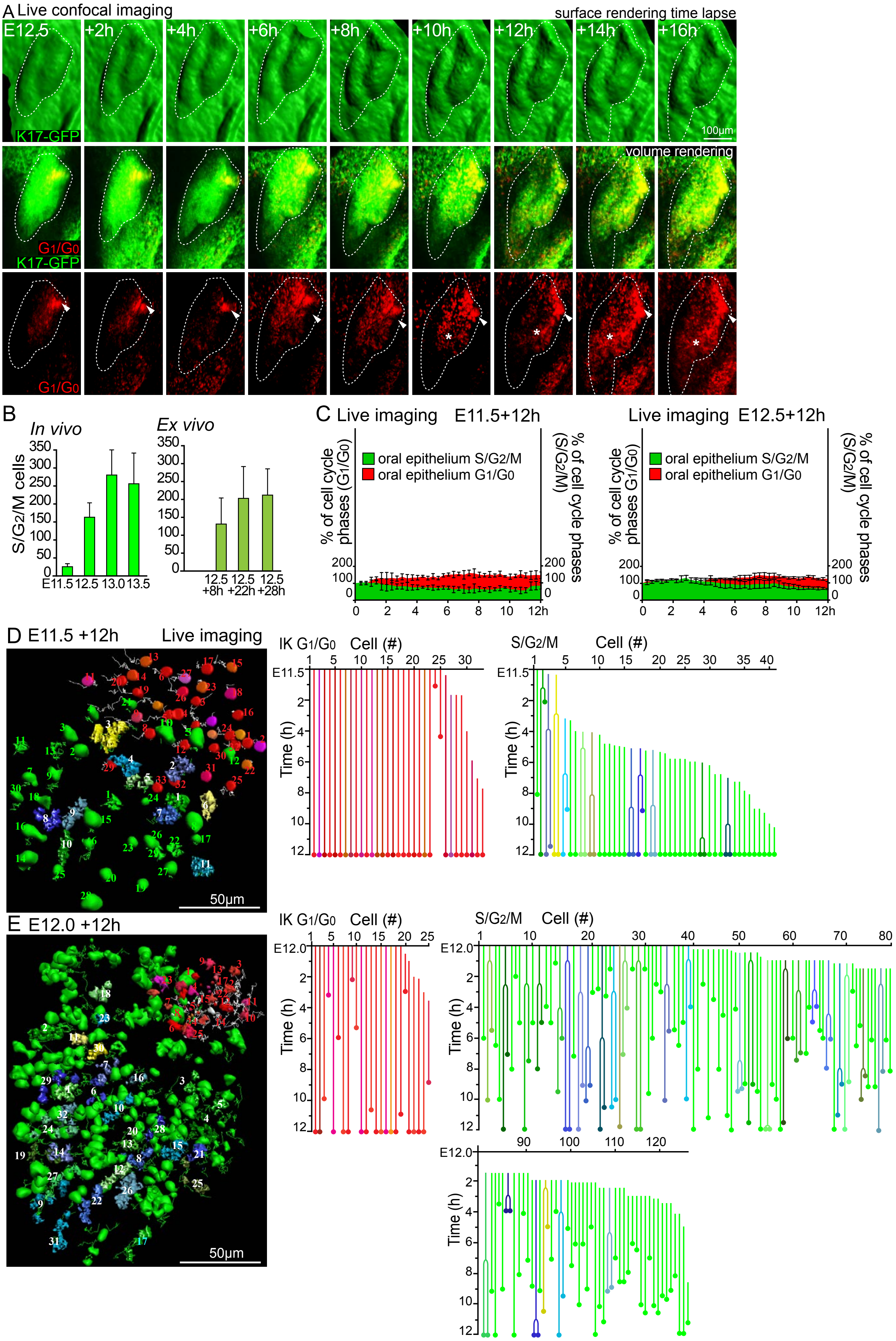

Development • Supplementary information

Related to Figure 2.

Figure S2. Tooth epithelial cell populations contribute differentially to the growing bud

(A) Still images of live tissue confocal microscopy time lapse of the Fucci G_1/G_0 reporter (to visualize the molar IK cells, red) and K17-GFP reporter (to visualize the borders and shape of the epithelial bud, green) in the developing first mandibular molar from E12.5 and consecutive 16 hours. This showed that the IK stays an integral part of the developing bud, and does not comprise emerging and regressing vestigial tooth structures. (B) Quantification of proliferating $S/G_2/M$ cell number in developing molar epithelium *in vivo* ($N_{E11.5}$=7, $N_{E12.5}$=8, $N_{E13.0}$=4, $N_{E13.5}$=5, mean±SD) was similar to explants grown *ex vivo (*$N_{E12.5+8h}$=4, $N_{E12.5+22h}$=7, $N_{E12.5+28h}$=4, mean±SD). (C) Quantification of cell cycle phases in oral epithelial cells of Fucci G_1/G_0 and $S/G_2/M$ whole mount live imaging samples, corresponding to the tooth epithelium area, from E11.5 and the following 12 hours (N=5, mean±SEM) and similarly E12.5+12h (N=3, mean±SEM). (D) Cell division kinetics and single cell fates: Surface rendering composite still image of a live tissue whole mount time lapse E11.5+12h. Tracing the contribution of individual G_1/G_0 (red numbers) and $S/G_2/M$ (green numbers) cells originating from various positions in the placode/bud, and cell divisions (white numbers). Divisions (where cytokinesis was observed) in surface rendering shown mother and daughter cells respectively color coded. Line graph shows the single cell fates and contribution of individual G_1/G_0 and $S/G_2/M$ cells to the molar from E11.5+12h. (F) Surface rendering composite still image of a live tissue whole mount time lapse E12.0+12h and line graph showing the single cell fates and contribution of individual G_1/G_0 and $S/G_2/M$ cells.

Development • Supplementary information

Supplemental Figure S3. (related to Figure 3.)

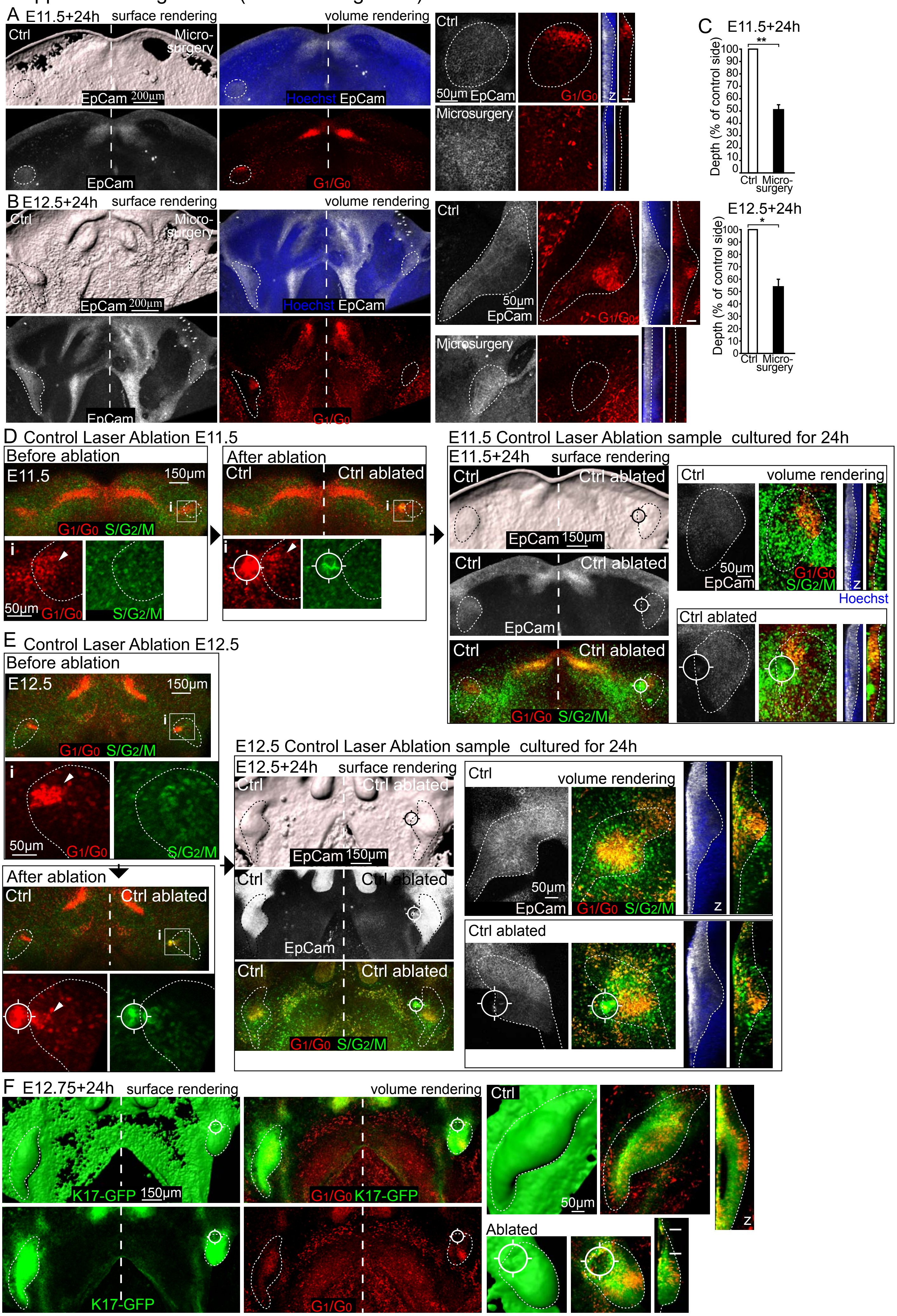

Related to Figure 3.

Figure S3. Microsurgical removal of the IK abrogates molar bud growth

Confocal fluorescence images of Fucci G_1/G_0 (red) whole mount explant cultures, EpCam immunofluorescence staining (grey, epithelium: dotted line), nuclei Hoechst (blue). (A) The molar epithelial placode was microsurgically removed at E11.5 and the tissue cultured for 24h. No G_1/G_0 condensate was present in the diastema and the epithelium remained flat while the control bud invaginated normally. (B) Microsurgical removal of the IK at E12.5 similarly arrested molar growth. The control side development proceeded normally to bud stage with the emerging pEK present. (C) Quantification of epithelial bud depth on the control side compared to epithelium depth in the respective area on the side where the IK was microsurgically removed in E11.5+24h and E12.5+24h explants ($N_{E11.5+24h}$=8, $N_{E12.5+24h}$=7, mean±SEM, Mann Whitney U, $p \leq 0.05$*, $p \leq 0.01$**). (D) Control laser ablation, in the early placode stage (E11.5), of an area adjacent to the IK G_1/G_0 cells leaving the signaling center intact permitted normal progression of tooth development to bud stage when cultured for 24h. Normal proliferation pattern was seen in teeth, and oral epithelium, and mesenchyme visualized with the Fucci $S/G_2/M$ reporter (green) in control ablated samples. The laser power in all ablation experiments was adjusted so that the ablation only affected the epithelium and not the underlying mesenchyme: Hoechst nuclear staining, in addition to unaffected proliferation pattern, showed intact mesenchyme. (E) Similarly, control ablation at early bud stage E12.5 permitted normal progression of molar tooth development and the emergence of the pEK. (F) Laser ablation of the IK in a later stage, at E12.75, arrested bud growth. We, however, observed a small cluster of G_1/G_0 cells in the bottom of bud, in the epithelium mesenchyme interface, 24h after ablation, likely corresponding to local mesenchyme induced pEK fate in the cells.

Development • Supplementary information

Supplemental Figure S4. (related to Figure 4.)

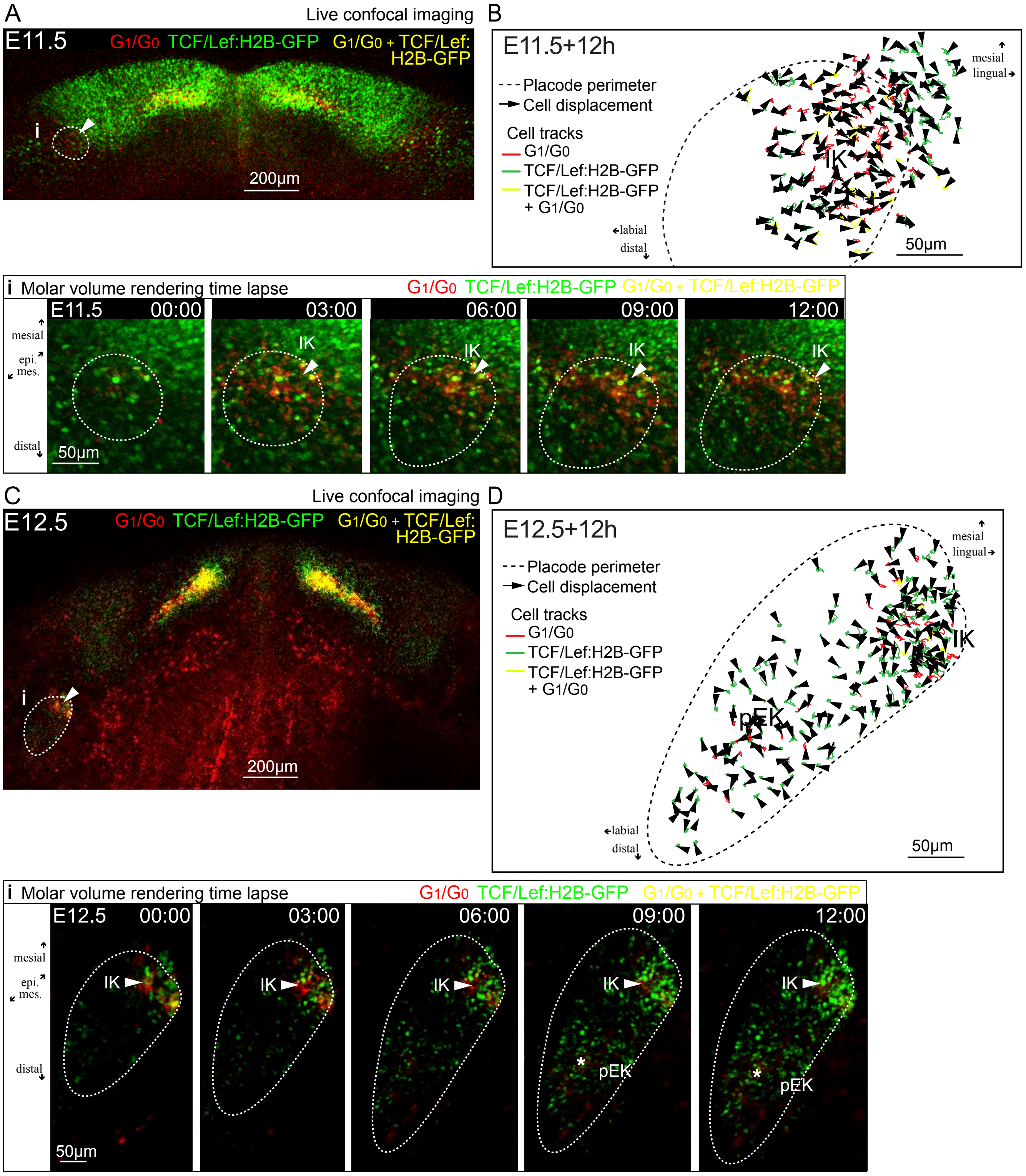

Development • Supplementary information

Related to Figure 4.

Figure S4. TCF/Lef:H2B-GFP reporter confirms that the IK remains an integral part of the tooth and does not contribute cells to the pEK

(A) Fucci G_1/G_0 (red) and TCF/Lef:H2B-GFP (green), Fucci G_1/G_0 + TCF/Lef:H2B-GFP (yellow), closeup inset (i). Still images of a live tissue time lapse confocal imaging from placode stage at E11.5 (whole mandible) and molar closeup E11.5+12h (E11.5, +3h, +6h, +9h and +12 hours). (B) Tracks and displacement vectors of individual Fucci G_1/G_0, double positive, and TCF/Lef:H2B-GFP cells in the E11.5+12h molar culture. (B) Still images of a Fucci G_1/G_0 and TCF/Lef:H2B-GFP reporter live imaging from early bud stage at E12.5 to +12h. (D) Tracks and displacement vectors of individual Fucci G_1/G_0, double positive, and TCF/Lef:H2B-GFP cells in the E12.5+12h molar culture.

Development • Supplementary information

Supplementary Figure S5. (related to Figure 6.)

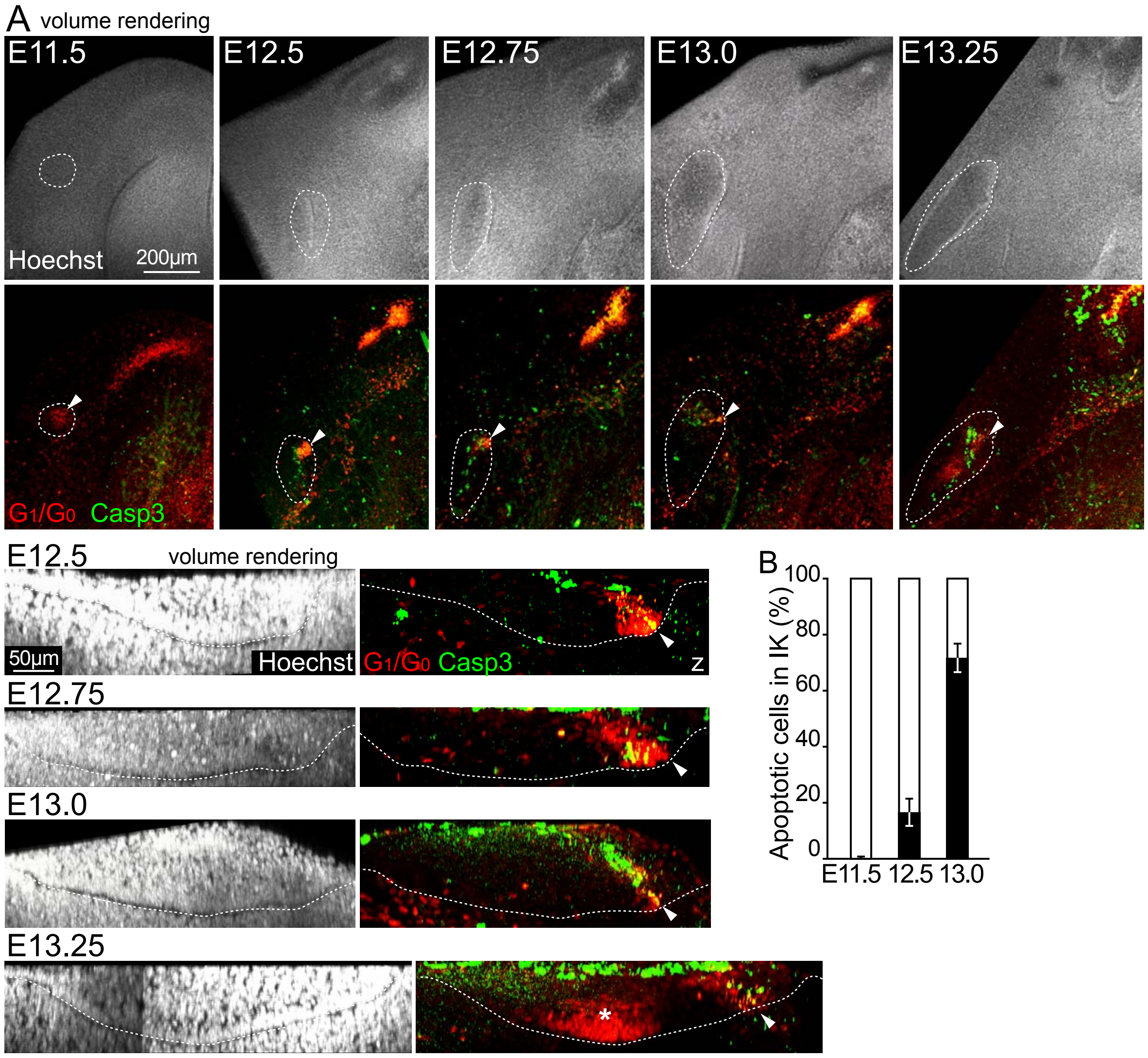

Related to Figure 6.

Figure S5. Apoptosis as a silencing mechanism of the molar IK signaling center

(A) Whole mount confocal fluorescence images of Fucci G_1/G_0 (red), cleaved caspase 3 immunofluorescence staining for apoptotic cells (Casp3, green). Epithelial bud (dotted line), IK (arrowhead) and presumptive pEK (asterisk), volume rendering close-up from side view of the molar (z). (B) Quantification of Casp3+ nuclei in the molar IK (N=12, mean±SEM).

Development • Supplementary information

Supplemental Figure S6. (related to Figure 7.)

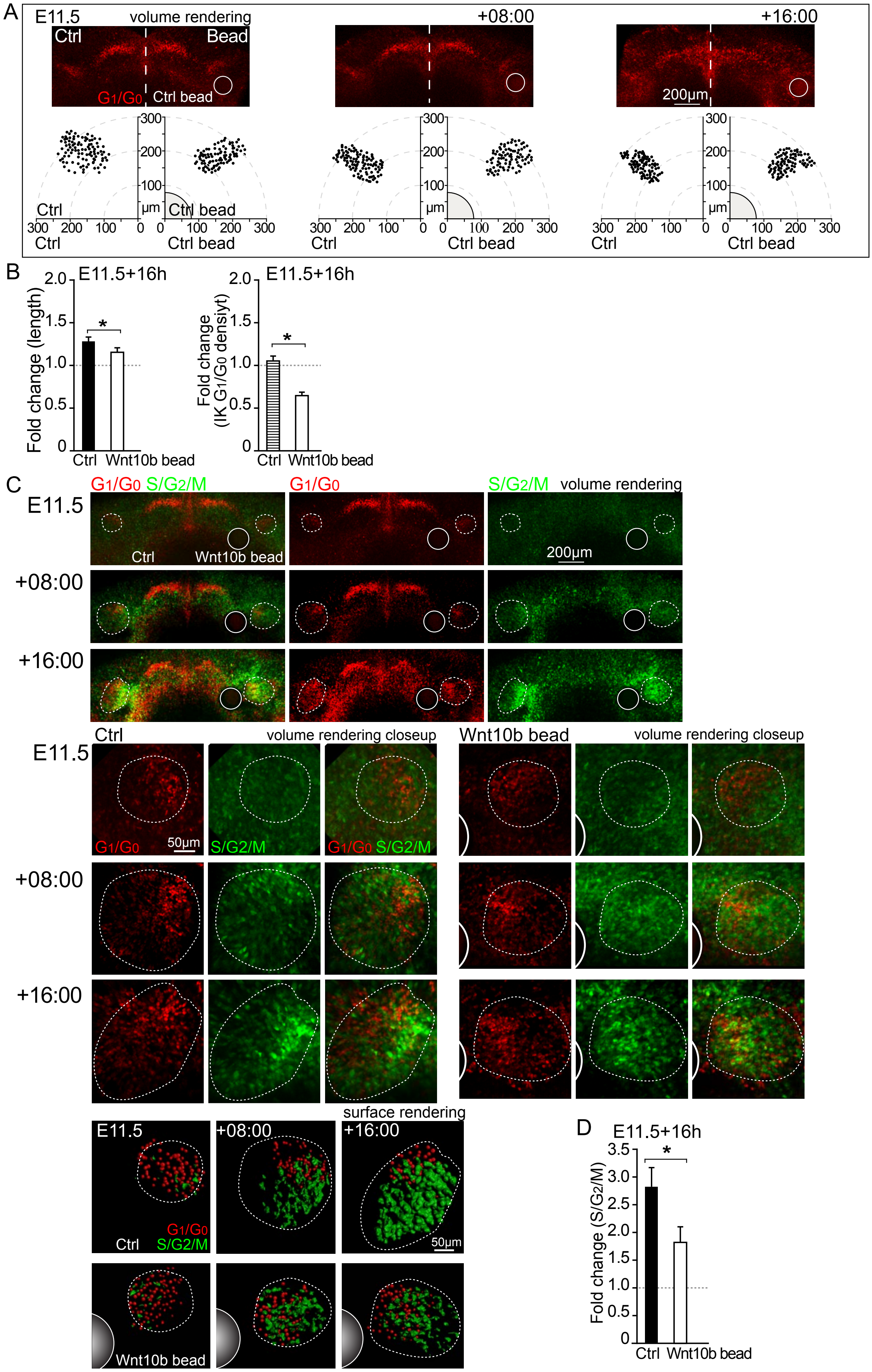

Development • Supplementary information

Related to Figure 7.

Figure S6. Exogenous Wnt10b alters cell population distribution in developing molars

(A) A control bead was placed next to the IK (visualized with Fucci G_1/G_0 reporter) distally to the placode and explant was imaged at E11.5, after 8h, and 16h. Morphogenesis and IK condensation did not differ from the untreated tooth on the control bead side. (B) Quantification of bud length and IK G_1/G_0 cell density (fold change over start of the treatment) in E11.5+16h samples treated with a recombinant Wnt10b protein releasing bead or control (N=10, mean±SEM, Mann Whitney U, $p \leq 0.05$*). (C) Confocal fluorescence images of Fucci G_1/G_0 (red), $S/G_2/M$ (green) whole mount explant cultures, tooth epithelium (dotted line), bead (closed circle). (D) Quantification shows decrease in proliferation (number of $S/G_2/M$ cells, fold change over start of the treatment) at E11.5+16h in Wnt10b bead treated molar buds compared to controls (N=6, mean±SEM, Mann Whitney U, $p \leq 0.05$*).

Development • Supplementary information

Supplemental Figure S7. (related to Materials and Methods)

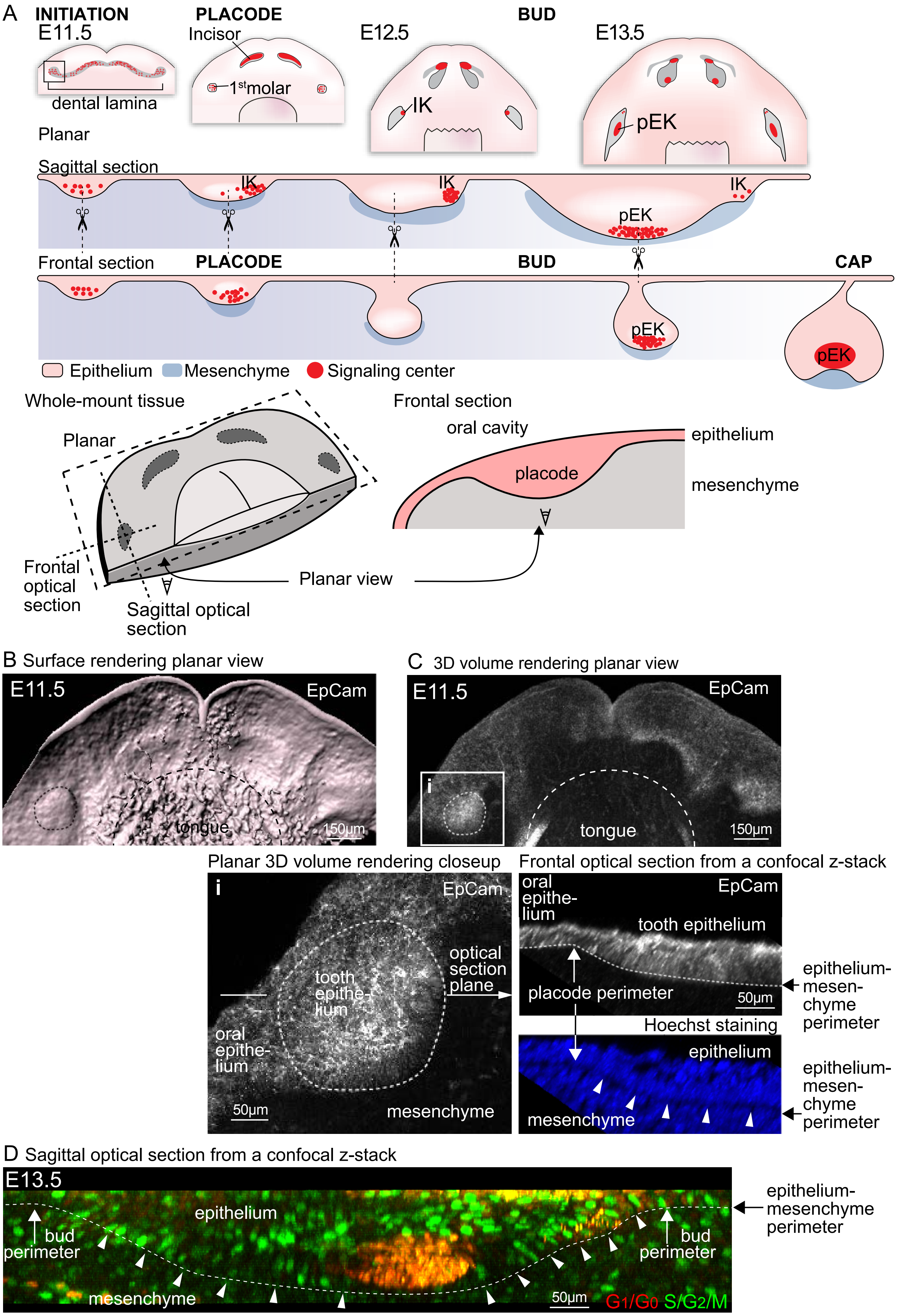

Development • Supplementary information

Related to Materials and Methods

Figure S7. Viewpoints and 3D rendering of tissue

(A) Schematic representation of different viewpoints for the 3D volume rendering of a planar confocal z-stack and optical sectioning. The planar view is presented in all figures from the mesenchyme toward the epithelium. Frontal and sagittal optical sectioning orientation marked with dotted lines. (B) Example of a surface rendering created by thresholding in Imaris from a confocal z-stack of the tissue. The EpCam staining, shown here in a E11.5 whole-mount mandible, is specific to the epithelium and 3D surface rendering visualizes the epithelium shape. The right molar placode is marked with a dotted line (short dots). The tongue has been removed, but the place of the tongue is indicated with a dotted line (longer dots). (C) The overview and close-up (i) of the 3D volume rendering of the same E11.5 whole mount mandible as in B. This rendering shows the whole voxel volume of the z-stack. Volume rendering efficiently visualizes the borders of the placode: The perimeter of the tooth and oral epithelium is marked with a dotted line in the EpCam volume rendering planar view. In the frontal optical section the epithelium perimeter facing the mesenchyme is marked with a dotted line and in the Hoechst stained view, showing nuclei, this epithelium-mesenchyme border is marked with arrowheads. Tooth placode perimeter facing the oral epithelium is marked with an arrow. (D) An example of a sagittal optical section of a z-stack volume rendering of the Fucci cell cycle reporter that visualizes individual nuclei. The reporter is expressed both in the epithelium and mesenchyme and, therefore, border of the tissue is readily detectable with morphological characteristics, similarly as with the Hoechst staining.

Table S1. Experimental conditions, sample size, and analysis

Click here to download Table S1

Development • Supplementary information

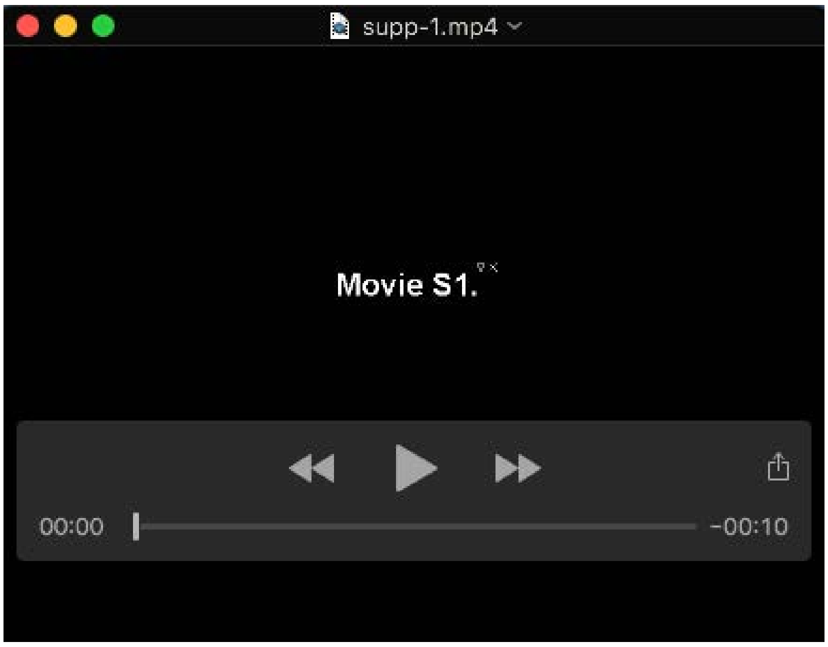

Related to Figure 2.

Movie 1. Tracking individual cell G1 and S/G2/M fates in a Fucci cell cycle reporter E11.5+12h molar placode by live tissue confocal imaging shows placode IK cells stay in G1 phase

Fluorescence confocal microscopy time-lapse of an embryonic mouse E11.5 whole-mount mandible explant imaged for 12h. Image stacks were taken at 20min intervals, and the playback speed here is five frames per second. The tracking of individual cell fates from the placode stage is shown as a surface rendering of the Fucci cell cycle indicator: the contribution of individual G_1/G_0 phase cells in the placode and initiation knot are shown color coded in red hues and $S/G_2/M$ cells in green. Individual cell divisions of mother cells and their daughters are color coded in blue, green and yellow shades, respectively. IK G_1/G_0 cells in the placode did not re-enter the cell cycle. In contrast neighboring cells, distally from the IK, frequently entered $S/G_2/M$ phase and cell divisions contributed to invagination locally.

Development • Supplementary information

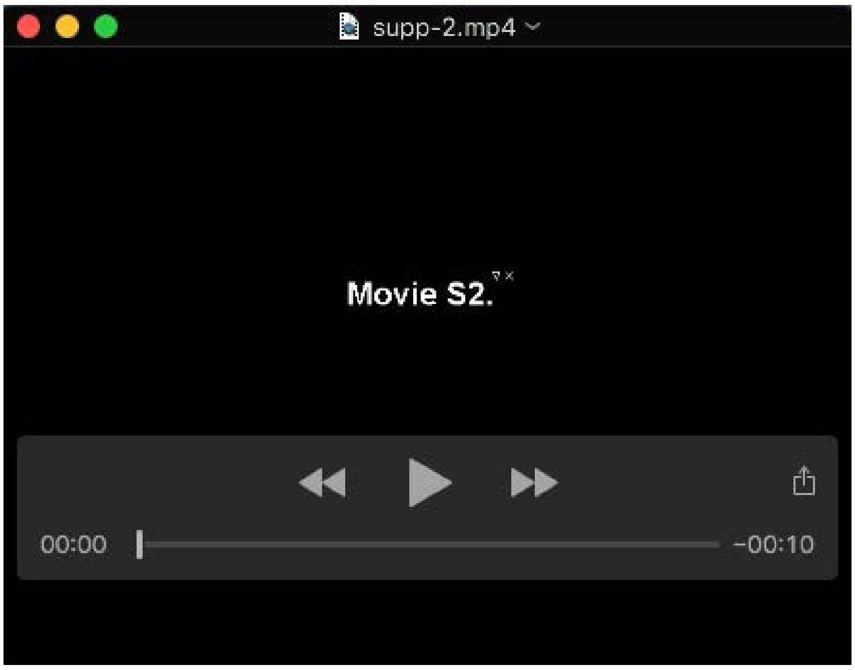

Related to Figure 2.

Movie 2. The IK remains an integral part of the growing tooth bud as shown in Fucci cell cycle reporter E12.0+12 h molars by confocal live tissue imaging

Fluorescence confocal microscopy time-lapse of a Fucci cell cycle indicator E12.0 whole-mount mandible explant imaged for 12h. Image stacks were taken at 20min intervals, and the playback speed here is five frames per second. The tracking of individual cell fates during the early bud invagination, is shown as a surface rendering: G_1/G_0 phase cells are shown in red hues and S/G2/M cells in green. Individual cell divisions of mother cells and their daughters are color coded in blue, green and yellow shades, respectively. IK cells remained in G_1/G_0 phase and remained an integral part of the growing tooth. S/G_2/M phase cells and cell divisions in both basal and suprabasal populations contributed to invagination and growth throughout the bud.

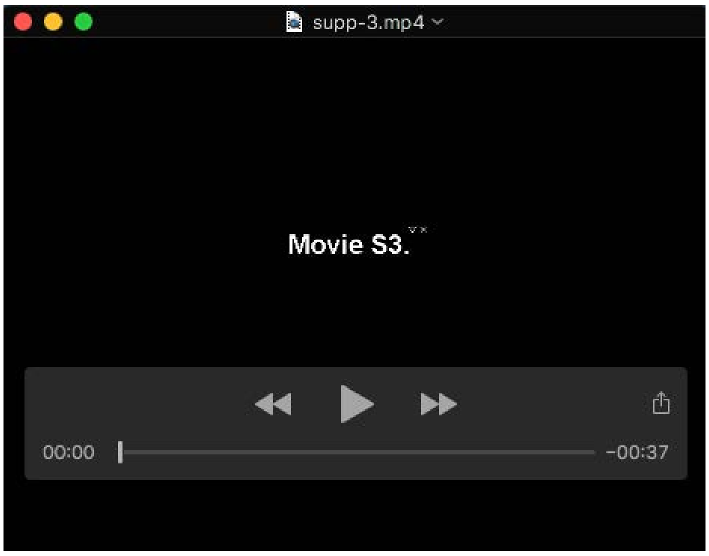

Related to Figure 4.

Movie 3. Live imaging of Fucci cell cycle reporter shows that IK cells rearrange dynamically in the E11.5+12h molar placode

Fluorescence confocal microscopy time-lapse of an embryonic mouse E11.5 whole-mount mandible explant imaged for 12h, showing the contribution of cell cycle stages to molar placode and initial budding morphogenesis on a high single cell resolution. Image stacks were taken at 20min intervals, and the playback speed here is five frames per second. The movie shows a volume rendering of cell cycle indicator Fucci G_1/G_0 nuclei (red) and $S/G_2/M$ (green). First, an overview of the mandible at the start of imaging seen from the mesenchymal side toward the epithelium, then a close up of the developing molar placode (IK, open circle) followed by both channels merged and separately. Individual cell tracks are shown as a dragon tail rendering showing a subset of twenty subsequent points in each track, IK G_1/G_0 (red), $S/G_2/M$ (green). The IK G_1/G_0 cells moved toward the mesial front area of the bud. The IK cells stayed in G_1/G_0 phase and drove proliferation locally in the adjacent cells, posterior to the knot, to initiate the invagination of the epithelium. The bud $S/G_2/M$ cells showed little movement.

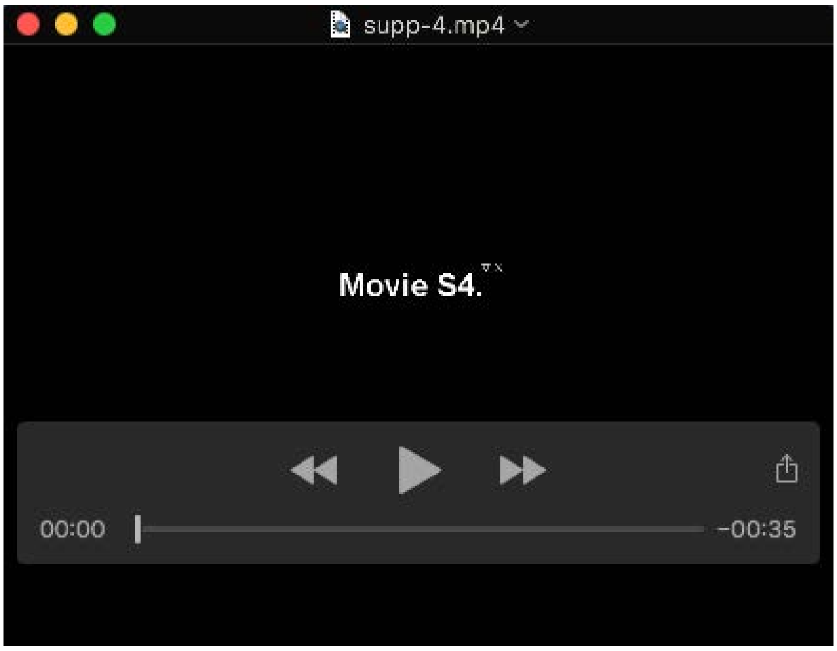

Related to Figures 4, 6 and S4.

Movie 4. Live imaging shows TCF/Lef:H2B-GFP and Fucci G1 marker expressing cells are closely juxtaposed and exhibit differential movement patterns in E11.5+12h molar placodes

Fluorescence confocal microscopy time-lapse of E11.5+12h Fucci G_1/G_0 and TCF/Lef:H2B-GFP reporters. Image stacks were taken at 20min intervals, and the playback speed here is five frames per second. The movie shows a volume rendering of Fucci G_1/G_0 nuclei (red) and TCF/Lef:H2B-GFP canonical Wnt signaling reporter (green). First an overview of the mandible at the start of imaging seen from the mesenchymal side toward the epithelium, then a close up of the developing molar placode (IK, open circle) followed by both channels merged and separately. Individual cell tracks are shown as a dragon tail rendering showing a subset of ten subsequent points in each track, IK G_1/G_0 (red), TCF/Lef:H2B-GFP (green), both reporters (yellow). The molar placode IK G_1/G_0 cells specifically localized to the peripheral border formed by dental lamina cells with high TCF/Lef:H2B-GFP reporter expression (Wnt^{Hi} cells). Increasing numbers of G_1/G_0 cells were recruited to the IK with directional movement, toward the dental lamina Wnt^{Hi} cells. Dental lamina Wnt^{Hi} cells remained mostly localized.

Development • Supplementary information

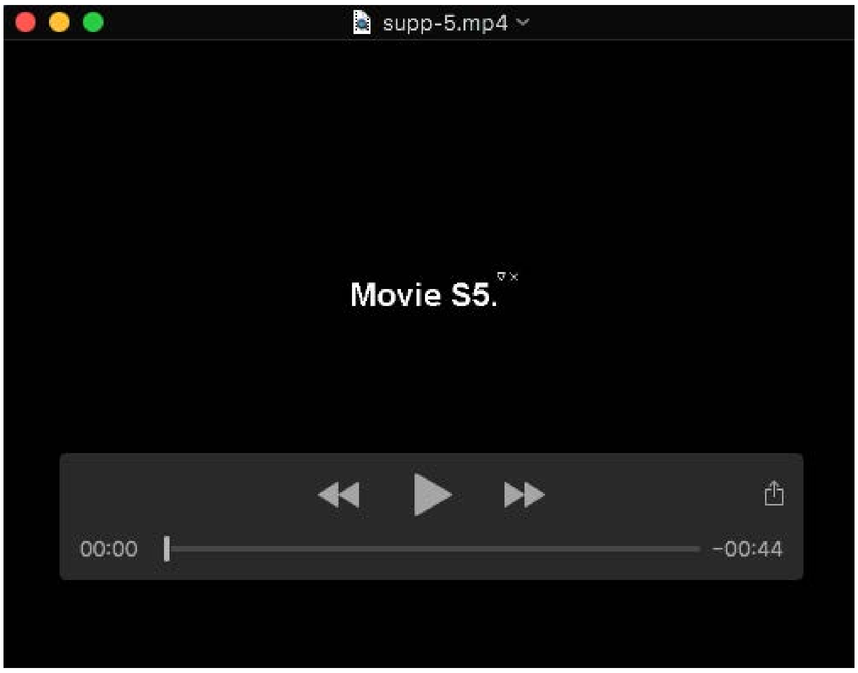

Related to Figures 3 and 4

Movie 5. Live imaging of E12.5+12h Fucci reporter shows that IK G1 cells do not contribute clonally to the primary EK in the molar

Fluorescence confocal microscopy time-lapse of E12.5+12h Fucci explant, showing the contribution of cell cycle stages at bud stage and initiation of the pEK. Image stacks were taken at 20min intervals, and the playback speed here is five frames per second. The movie shows volume rendering of Fucci G_1/G_0 nuclei (red) and $S/G_2/M$ (green) overview of the mandible at the start of imaging (initial molar bud outlined white). In the close up of the molar IK is marked with an open circle and the location where the pEK will be initiated (marked with a closed circle). Time lapse shows volume rendering of both channels merged and separately. In track view the perimeter of the mature bud is outlined in white. Individual cell tracks are shown as a dragon tail rendering showing a subset of ten subsequent points in each track, IK G_1/G_0 (red), pEK G_1/G_0 (magenta), $S/G_2/M$ (green). A wave of cell proliferation contributed to rapid bud growth while the IK cells stayed in G_1/G_0 in the mesial part of the bud. The pEK G_1/G_0 cells were initiated *de novo* deep in the tip of the invaginating bud with no clonal contribution from the IK.

Development • Supplementary information

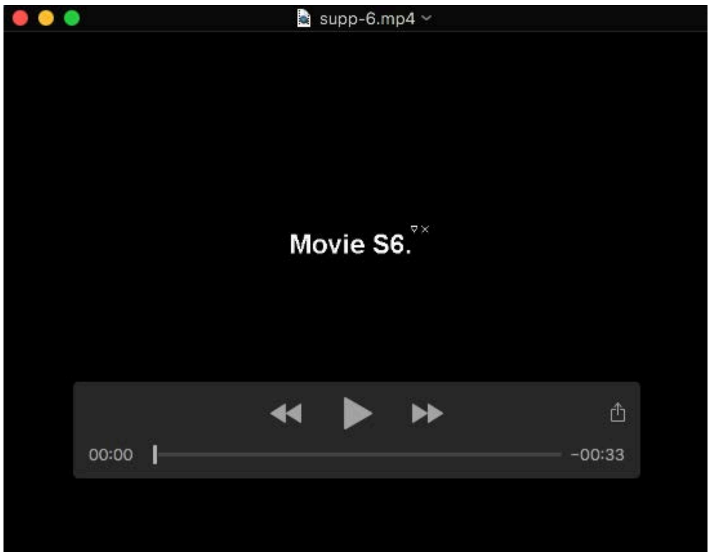

Related to Figure 4. and S4.

Movie 6. Live imaging of Fucci and TCF/Lef:H2B-GFP reporters shows independent IK and pEK signaling centers regulating morphogenesis in E12.5+12h molar

Fluorescence confocal microscopy time-lapse of Fucci G_1/G_0 and TCF/Lef:H2B-GFP E12.5+12h whole mount explant. The movie shows volume rendering of Fucci G_1/G_0 nuclei (red) and TCF/Lef:H2B-GFP canonical Wnt signaling reporter (green) visualizing the IK and emerging pEK signaling centers. Image stacks were taken at 20 min intervals, and the playback speed here is five frames per second. An overview of a volume rendering of the mandible at the start of imaging shows both channels merged and the molar bud is outlined in white. In close up view of the molar IK position is marked with an open circle and the location where the pEK is later initiated is marked with a closed circle. Time lapse shows volume rendering of both channels merged and separately. In track view the perimeter of the mature bud is outlined in white. Individual cell tracks are shown as a dragon tail rendering showing a subset of ten subsequent points in each track, IK G_1/G_0 (red), pEK G_1/G_0 (magenta), high intensity TCF/Lef:H2B-GFP (Wnt^{Hi}, green), double positive (Wnt^{Hi}+ Fucci G_1/G_0, yellow). Initially at E12.5, Wnt^{Hi} cells surrounded the G1 cells in the IK. TCF/Lef:H2B-GFP positive cells appeared throughout the bud with increase in signal intensity in the prospective pEK region followed by emergence of first G1 cells. Tracking of individual G_1/G_0, Wnt^{Hi}, and double positive cells showed that none of these cell populations from the IK contributed clonally to the pEK; the Wnt^{Hi} and G_1/G_0 cells in the pEK region appeared *de novo*.
